# Single-Cell Profiling of Ebola Virus Disease *In Vivo* Reveals Viral and Host Dynamics

**DOI:** 10.1016/j.cell.2020.10.002

**Published:** 2020-11-25

**Authors:** Dylan Kotliar, Aaron E. Lin, James Logue, Travis K. Hughes, Nadine M. Khoury, Siddharth S. Raju, Marc H. Wadsworth, Han Chen, Jonathan R. Kurtz, Bonnie Dighero-Kemp, Zach B. Bjornson, Nilanjan Mukherjee, Brian A. Sellers, Nancy Tran, Matthew R. Bauer, Gordon C. Adams, Ricky Adams, John L. Rinn, Marta Melé, Stephen F. Schaffner, Garry P. Nolan, Kayla G. Barnes, Lisa E. Hensley, David R. McIlwain, Alex K. Shalek, Pardis C. Sabeti, Richard S. Bennett

**Affiliations:** 1 Department of Systems Biology, Harvard Medical School, Boston, MA 02115, USA; 2 FAS Center for Systems Biology, Department of Organismic and Evolutionary Biology, Harvard University, Cambridge, MA 02138, USA; 3 Broad Institute of MIT and Harvard, Cambridge, MA 02142, USA; 4 Harvard-MIT Division of Health Sciences and Technology, Massachusetts Institute of Technology, Cambridge, MA 02142, USA; 5 Harvard Program in Virology, Harvard Medical School, Boston, MA 02115, USA; 6 Integrated Research Facility, Division of Clinical Research, National Institute of Allergy and Infectious Diseases, National Institutes of Health, Frederick, MD 21702, USA; 7 Department of Chemistry, Institute for Medical Engineering and Sciences (IMES), and Koch Institute for Integrative Cancer Research, MIT, Cambridge, MA 02142, USA; 8 Ragon Institute of MGH, Harvard, and MIT, Cambridge, MA 02139, USA; 9 Department of Pathology, Stanford University, Stanford, CA 94305, USA; 10 Trans-NIH Center for Human Immunology, Autoimmunity, and Inflammation, National Institutes of Health, Bethesda, MD 20814, USA; 11 BioFrontiers Institute, University of Colorado Boulder, Boulder, CO 80303, USA; 12 Life Sciences Department, Barcelona Supercomputing Center, Barcelona, Catalonia 08034, Spain; 13 Department of Immunology and Infectious Diseases, Harvard T.H. Chan School of Public Health, Harvard University, Boston, MA 02115, USA; 14 MRC-University of Glasgow Centre for Virus Research, Glasgow G61 1QH, UK; 15 Howard Hughes Medical Institute, Chevy Chase, MD 20815, USA

**Keywords:** Ebola virus, single-cell, scRNA-Seq, Seq-Well, CyTOF, monocytes, bystander cells, interferon, viral tropism, host-virus interactions

## Abstract

Ebola virus (EBOV) causes epidemics with high mortality yet remains understudied due to the challenge of experimentation in high-containment and outbreak settings. Here, we used single-cell transcriptomics and CyTOF-based single-cell protein quantification to characterize peripheral immune cells during EBOV infection in rhesus monkeys. We obtained 100,000 transcriptomes and 15,000,000 protein profiles, finding that immature, proliferative monocyte-lineage cells with reduced antigen-presentation capacity replace conventional monocyte subsets, while lymphocytes upregulate apoptosis genes and decline in abundance. By quantifying intracellular viral RNA, we identify molecular determinants of tropism among circulating immune cells and examine temporal dynamics in viral and host gene expression. Within infected cells, EBOV downregulates *STAT1* mRNA and interferon signaling, and it upregulates putative pro-viral genes (e.g., *DYNLL1* and *HSPA5*), nominating pathways the virus manipulates for its replication. This study sheds light on EBOV tropism, replication dynamics, and elicited immune response and provides a framework for characterizing host-virus interactions under maximum containment.

## Highlights

• Interferon response is suppressed in infected cells but activated in bystander cells
• EBOV represses antiviral genes and upregulates pro-viral genes in infected cells
• Proliferative CD14^–^ CD16^–^ monocyte precursors expand in circulation during EVD
• Identification of expression markers of EBOV tropism for circulating cells *in vivo*

## Graphical Abstract

**Figure:**
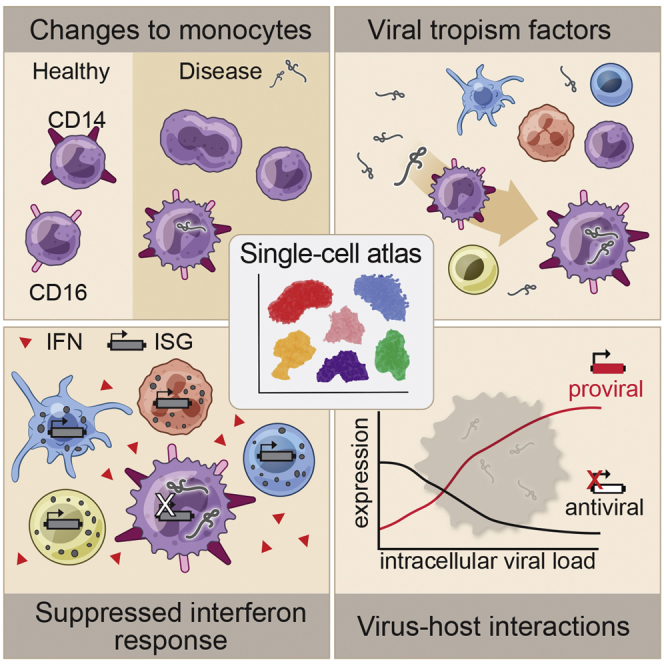

## Teaser

Single-cell profiling of circulating immune cells during Ebola virus (EBOV) infection in non-human primates resolves molecular correlates of viral tropism, characterizes replication dynamics within infected cells, and distinguishes expression changes that are mediated by viral infection from those due to cytokine signaling.

## Introduction

Ebola virus (EBOV) is among the world’s most lethal pathogens, with estimated case fatality rates of 40%–66% in recent epidemics (Lo et al., 2017; Ilunga Kalenga et al., 2019). EBOV infection in humans causes Ebola virus disease (EVD), characterized by fever, malaise, muscle aches, and gastrointestinal distress, rapidly progressing to coagulopathy, shock, and multi-organ failure (Malvy et al., 2019). While recently developed vaccines (Kennedy et al., 2017) and monoclonal antibody therapeutics (Mulangu et al., 2019) have shown great promise, case fatality rates in treated patients still exceed 30%, highlighting the need for further research into EVD.

Studies of EVD pathogenesis face numerous logistical challenges that have limited their scope. Experiments with live EBOV require maximum containment (biosafety level 4 [BSL-4]), restricting them to a few specialized research facilities. *In vivo* studies are especially challenging: human EVD is difficult to study during deadly outbreaks in resource-limited settings, necessitating animal models. Commonly used laboratory mice do not display key features of human EVD when exposed to naturally occurring EBOV (Bray, 2001; Geisbert et al., 2002). While other small animal models such as ferrets mostly recapitulate human EVD (Cross et al., 2016, 2018; Kozak et al., 2016), they are genetically distant from primates and lack the primate-specific genotype for *NPC1* (Diehl et al., 2016; Urbanowicz et al., 2016), the key cellular receptor for EBOV entry (Carette et al., 2011; Côté et al., 2011). EVD in nonhuman primates (NHPs) most closely resembles human EVD (Bennett et al., 2017; Geisbert et al., 2015; St Claire et al., 2017), but NHP studies are often limited to small sample sizes.

The two main approaches to studying EVD—analyzing infected cells in culture and infected animals *in vivo—*have revealed important, if somewhat contradictory, aspects of EBOV’s impact on the immune system. In culture, EBOV infects myeloid cells, potently inhibiting both production of type 1 interferon (Basler et al., 2003; Gupta et al., 2001) and signal transduction downstream of interferon receptors (Harcourt et al., 1999; Kash et al., 2006). Under-activation of this key innate antiviral response hinders the activation of the adaptive immune system by antigen-presenting cells (Bosio et al., 2003; Lubaki et al., 2013), a key determinant of fatal outcomes (Baize et al., 1999). EVD *in vivo*, by contrast, is characterized by high fever and dramatic upregulation of hundreds of interferon-stimulated genes (ISGs) (Caballero et al., 2016; Liu et al., 2017), in response to dozens of inflammatory cytokines (Reynard et al., 2019; Wauquier et al., 2010), suggesting that an aberrant over-activation of innate and adaptive immunity underlies much of EVD pathology (Geisbert et al., 2003a, 2003b).

High-throughput single-cell technologies, such as single-cell RNA-sequencing (scRNA-seq) and protein quantification by CyTOF (Bendall et al., 2011), have enabled the analysis of viral infection at unprecedented resolution (Hamlin et al., 2017; Hein and Weissman, 2019; Newell et al., 2012; O’Neal et al., 2019; Russell et al., 2018, 2019; Steuerman et al., 2018; Zanini et al., 2018a, 2018b; Zhao et al., 2020). These methods quantify the cell-type composition and expression programs of individual cells—signals that are obscured in bulk measurements. By quantifying viral RNA within cells, scRNA-seq allows comparison of gene expression between infected and uninfected (bystander) cells in a diseased host, giving a far more nuanced view of host and viral gene expression within infected cells. This approach can also disentangle direct effects of infection within a cell from the effects of the inflammatory cytokine milieu, which bulk approaches cannot easily do. However, to date, technical constraints have prevented use of these approaches in a BSL-4 setting: many scRNA-seq technologies require droplet generators and inactivation protocols not well suited to that environment, while CyTOF instruments involve high-volume exhaust and superheated components incompatible with BSL-4 installation (Logue et al., 2019). Thus, new protocols compatible with sample inactivation are needed.

Here, we describe the first BSL-4 investigation of a pathogen with high-dimensional single-cell technologies. We applied CyTOF and Seq-Well—a portable single-cell RNA-seq platform (Gierahn et al., 2017)—to a total of 90 peripheral blood mononuclear cell (PBMC) samples from 21 rhesus monkeys prior to, or during, lethal EBOV challenge *in vivo*. We further inoculated PBMCs with EBOV *ex vivo* and profiled their gene expression with Seq-Well. These data allow us to dissect host-virus interactions and comprehensively catalog changes in cell-type abundance and cell state over the course of EVD. Moreover, as EBOV has an RNA genome and transcribes polyadenylated mRNAs, we detected viral RNA within individual cells, allowing us to define EBOV tropism with high resolution and identify EBOV-associated transcriptional changes in putative pro- and antiviral genes.

## Results

### Single-Cell Atlas of RNA and Protein Expression in Circulating Immune Cells from EBOV-Infected Rhesus Monkeys

**Figure 1:**
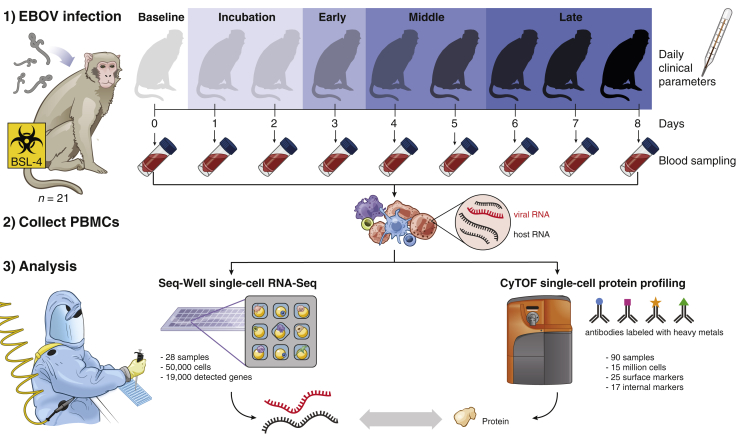
Study Design Under BSL-4 containment, we collected blood samples from a total of 21 rhesus monkeys at multiple days post-EBOV inoculation, extracted peripheral blood mononuclear cells (PBMCs), and profiled single-cell transcriptomes and 42 protein markers using Seq-Well and CyTOF. Seq-Well quantifies both host (black) and viral (red) RNA expression, allowing comparisons between infected and bystander cells. Daily clinical parameters (body temperature, clinical signs, and body weight) were also collected for each animal, and complete blood counts were obtained for each blood draw. See also Figure S1A and Table S1.

**Figure 2:**
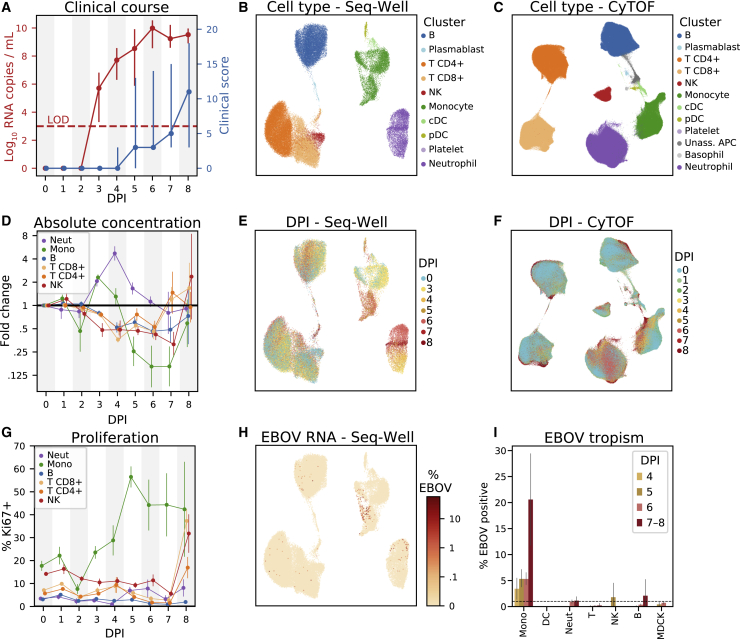
Changing Cell-Type Abundance, Proliferation Rate, and Infection Status during EVD (A) Time course of viral load (red, left y axis, log_10_ scale) and clinical score (blue, right y axis). Markers: mean; error bars: minimum and maximum; LOD, limit of detection by reverse transciption quantitative PCR. (B and C) Uniform Manifold Approximation and Projection (UMAP) embedding of Seq-Well (B) and CyTOF (C) data, colored by annotated cluster assignment. See also Figures S1 and S2, and Data S1. (D) Fold change (log_2_ scale) in the absolute abundance (cells/μL of whole blood) of each cell type relative to baseline based on CyTOF clusters. Error bars: mean ± 1 SE. See also Figures S3A and S3B. (E and F) UMAP embedding of Seq-Well (E) and CyTOF (F) data, colored by the day post-infection (DPI) on which each cell was sampled. (G) Percentage of Ki67-positive cells (CyTOF intensity >1.8) of each cell type. Error bars: mean ± 1 SE. See also Figures S3C and S3D. (H) UMAP embedding of Seq-Well data, colored by the percentage of cellular transcripts mapping to EBOV. (I) Percentage of infected cells by cell type based on Seq-Well. Dashed line: 1% false positive rate threshold for calling infected cells. Error bars: 95% CI on the mean based on 1,000 bootstraps. See also Figures S1H.

**Figure S1:**
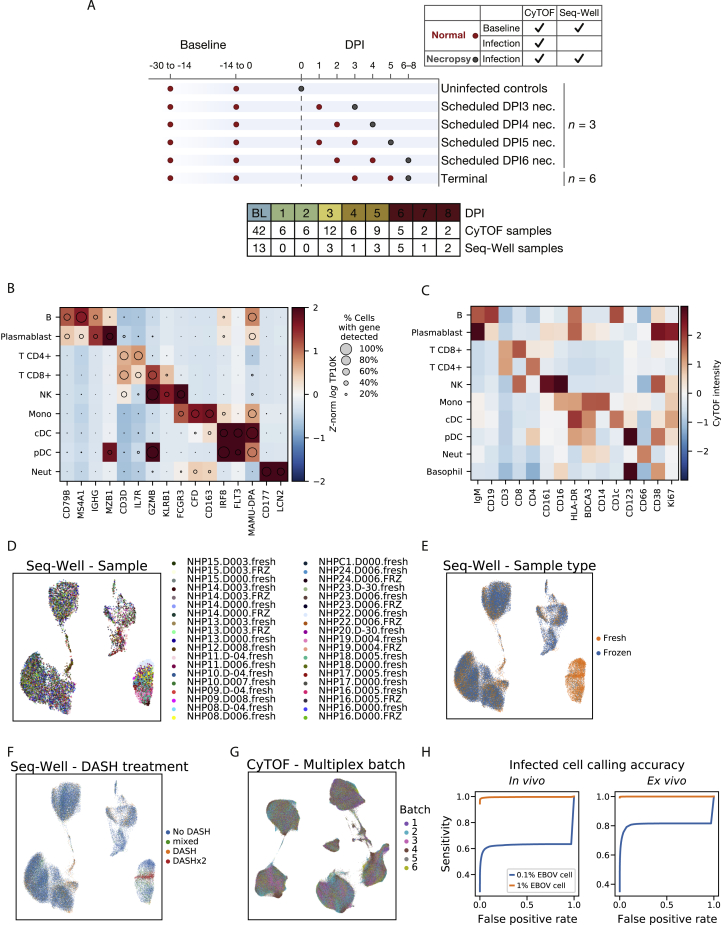
Cell-Type Markers for Seq-Well and CyTOF Clusters, Related to Figure 2 (A) Overview of study cohorts and blood draw timelines. Animals were grouped into cohorts with pre-scheduled necropsy times (at baseline, or day post infection [DPI] 3, 4, 5, 6 - n = 3 each), or allowed to progress until clinical score exceeded 10 (terminal), predetermined euthanasia criteria. Dots: scheduled blood draws for each cohort; red: intermediate (non-necropsy) draw; gray: draw that coincided with euthanasia and necropsy. Necropsy and baseline normal draws were used for Seq-Well and CyTOF, while intermediate post-infection draws were available only for CyTOF. (B) Expression profiles of cell-type marker genes (columns) for cell-type clusters (rows) based on the *in vivo* Seq-Well data. Circle area represents the percentage of cells in each group in which the gene was detected, and color denotes the average expression level (log_*e*_ TP10K). (C) Average expression (*Z*-normalized CyTOF intensity) profiles of cell-type marker genes (columns), for cell-type clusters (rows), based on the CyTOF data. (D) Uniform Manifold Approximation and Projection (UMAP) embedding of post-integration Seq-Well data, colored by the sample source (NHP, DPI, and whether the sample was loaded for Seq-Well without any freezing [.fresh] or was frozen with cryoprotectant and thawed prior to Seq-Well [.FRZ]). A maximum of 500 cells per sample is plotted to increase representation across samples. (E) UMAP embedding of Seq-Well data, colored by whether cells were processed fresh (orange) or after freeze/thaw (blue) prior to Seq-Well. (F) UMAP embedding of Seq-Well data, colored by depletion of abundant sequences by hybridization (DASH) treatment. We developed a DASH-based method to remove a PCR adaptor artifact from some Seq-Well sequencing libraries (STAR Methods), and performed this 0 times (No DASH, blue), 1 time (DASH, orange), or 2 times sequentially (DASHx2, red). For a few samples, we sequenced ‘No DASH’ and ‘DASH’ libraries and merged the reads (mixed, green). (G) UMAP embedding of batch-corrected CyTOF data, colored by the multiplex batch in which it was pooled and analyzed by CyTOF. (H) Receiver operating characteristic curves for identifying EBOV-infected cells. Estimates of sensitivity to detect an infected cell at various false positive rate thresholds *in vivo* (left) and *ex vivo* (right). Curves are estimated separately for a hypothetical viral load of 0.1% (blue line) and 1% (orange line).

To comprehensively profile EBOV-induced immune dysfunction *in vivo*, we collected peripheral immune cells prior to, and at multiple days post-infection (DPI), corresponding to different stages of acute EVD (Figure 1). Cohorts of ≥3 NHPs were sacrificed as baseline uninfected controls, at pre-defined DPI, or upon reaching humane euthanasia criteria. These cohorts were recently characterized for viral load, clinical score, blood chemistry (Bennett et al., 2020), and liver pathology (Greenberg et al., 2020). Viral load first became detectable in all NHPs on DPI 3, preceding detectable clinical signs (e.g., fever) by 1–2 days (Figure 2A). Clinical signs of EVD progressed until euthanasia criteria were reached at DPI 6–8. Cells collected at all time points were used for CyTOF, while cells collected at baseline and at sacrifice were also used for Seq-Well (Figure S1A).

**Figure S2:**
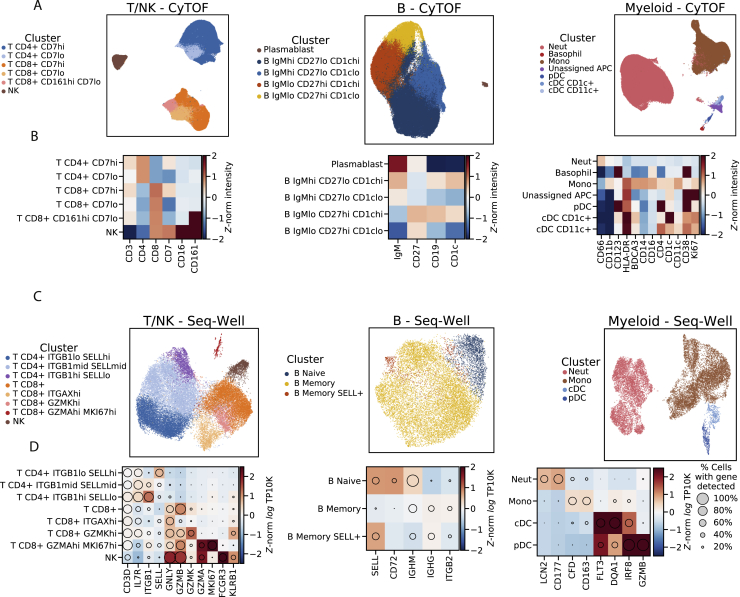
Identifying Cell Subtypes by Subclustering, Related to Figure 2 (**A**) UMAP embedding of broad cell-type clusters in the CyTOF data, colored by sub-cluster assignment (Neut: neutrophil, Mono: monocyte). (**B**) Average expression (*Z*-normalized CyTOF intensity) profiles of sub-clusters for marker genes based on CyTOF data. (**C**) UMAP embedding of broad cell-type clusters in the Seq-Well data, colored by sub-cluster assignment. (**D**) Expression profiles of sub-clusters for marker genes based on Seq-Well data. Circle area: percentage of cells in which the gene was detected; color: average expression level (*Z*-normalized log_*e*_ TP10K).

After standard quality control filters (STAR Methods), we obtained single-cell transcriptomes from ∼58,000 PBMCs and 42-protein CyTOF profiles from ∼15 million (M) PBMCs. We visualized these data with uniform manifold approximation and projection (UMAP) non-linear dimensionality reduction (Becht et al., 2018) (Figures 2B–2H). Unsupervised clustering of either the transcriptomes or a down-sampled set of 1.1 M protein profiles (STAR Methods) yielded clusters that could be readily identified from well-known RNA and protein markers as the major circulating immune cell types (Figures 2B, 2C, S1B, and S1C) and agreed well with manual gating (Data S1). After batch correction of the CyTOF data and integration of the transcriptomes (STAR Methods), samples were well distributed across cell-type clusters (Figures S1D–S1G) but separated by DPI (Figures 2E and 2F), indicating dynamic cell states over the disease course. By sub-clustering within cell types, we identified subtypes based on identifying marker genes (Figure S2).

### Cell-Type Abundance, Proliferation, and EBOV Infection Rates Vary throughout EVD

**Figure S3:**
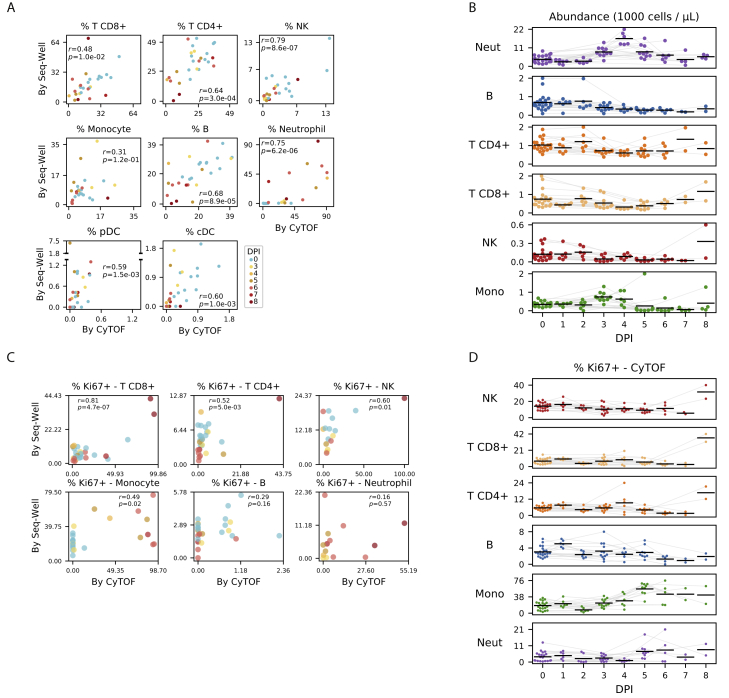
Estimates of Cell-Type Abundance and Proliferation over the Time Course, Related to Figure 2 (**A**) Scatterplot of the percentage of cells of each cell type in a sample, inferred from CyTOF (x axis) or Seq-Well (y axis), for several cell types (panels). Each dot represents a sample colored by DPI. Pearson correlation coefficients (*r*) and *p*-value are provided. (**B**) Estimates of the abundance of each cell type (rows) for each NHP (individual markers) in units of 1000 cells per μL of whole blood, based on integration of CyTOF and complete blood count (CBC) information. Black line: mean value of each DPI; gray lines: serial samples from the same NHP. (**C**) Scatterplots of the percentage of Ki67-positive cells in a sample inferred from CyTOF (x axis) or Seq-Well (y axis) for several cell types (panels). Each dot represents a sample colored by DPI. Cells with smoothed expression of *MKI67* (the gene coding for Ki67) > 0.1 are called Ki67-positive by Seq-Well. Cells with CyTOF intensity > 1.8 are called Ki67-positive by CyTOF. (**D**) Estimates of the percentage of Ki67-positive cells (CyTOF intensity > 1.8) of each cell type (rows) for each animal replicate (markers). Black line: mean value of each DPI; gray lines: serial samples from the same NHP.

In addition to the major PBMC cell types, a cluster of immature neutrophils emerged during EVD, marked by high gene expression of *CD177* and *SOD2*, and protein expression of CD66 and CD11b. Based on scRNA-seq, neutrophils increased from 0.2% of cells at baseline to 65.1% in late EVD (by CyTOF, from 9.3% to 49.8%; Figures S3A and S3B). Though neutrophils are typically removed during density-based PBMC isolations, immature neutrophils (band cells) are less dense than mature neutrophils and can be released from the bone marrow and co-isolate with PBMCs in infections, including during EVD (Eisfeld et al., 2017).

We quantified absolute abundance of each cell type during EVD by combining CyTOF or scRNA-seq data with complete blood counts (STAR Methods) (Bennett et al., 2020); both estimates were in general agreement (Figure S3A).

Neutrophil abundance increased from baseline by 5-fold by DPI 4, before returning to baseline levels in late EVD (p < 0.05 for DPI 3–4, p = 0.059 for DPI 5, Wilcoxon signed-rank test, Figures 2D and S3B). Lymphocyte abundance decreased, with natural killer (NK) cells declining 1 day before the other cell types (p < 0.05 on DPI 3–6 for B, NK, CD8^+^ T, and CD4^+^ T; except for CD4^+^ T on DPI 5); both observations are in agreement with previous NHP studies (Fisher-Hoch et al., 1985; Ebihara et al., 2011). All lymphocyte populations slowly recovered after DPI 4 (Figure S3B). Monocyte abundance initially increased >2-fold before declining precipitously between DPI 4 and 5.

Changes in circulating cell-type abundance could reflect cell proliferation, death, and movement into and out of bone marrow, lymph, and tissues. While we could not directly quantify death or movement between compartments, we estimated the fraction of dividing cells using the proliferation marker Ki67 in both the CyTOF and scRNA-seq data (Figure S3C). The fraction of Ki67^+^ monocytes increased from 17% at baseline to 56% at DPI 5 and remained > 40% thereafter (p = 1.1 × 10^–5,^ rank-sum test of DPI 5–8 versus baseline), suggesting increased proliferation (Figures 2G and S3D). The maximum on DPI 5 coincided with a decline in absolute abundance (Figures 2D and S3B), suggesting that losses due to extravasation or death dominate increases from proliferation in late EVD. By contrast, neutrophil proliferation remained roughly constant despite the dramatic changes in abundance, further evidence that immature neutrophils were released from the bone marrow during disease. Intriguingly, the fraction of dividing T and NK cells stayed relatively constant until increasing dramatically on DPI 8 for both NHPs that survived until then (RNA: Figure S3C; protein: Figure S3D, p = 0.022 rank-sum test of DPI 8 versus baseline for NK, CD8^+^ T, and CD4^+^ T). Proliferation is a core component of T cell mediated viral clearance but requires time for activated T cells to accumulate; the observation that significant T cell proliferation only occurred in the 2 NHPs at the latest DPI suggests that they may have begun to mount a T cell response.

Not all cell types support EBOV entry and replication. We used scRNA-seq to identify infected cells *in vivo* based on the presence of EBOV’s RNA genome and poly-adenylated mRNA (Figure 2H). We developed a statistical model to identify infected cells containing more EBOV reads than expected due to ambient RNA contamination (STAR Methods) and estimated a mean sensitivity of 51% when ≥0.1% of cellular transcripts derived from EBOV (false positive rate = 1%, Figure S1H). We also spiked uninfected Madin-Darby canine kidney (MDCK) cells into a subset of PBMC samples to serve as a negative control (Table S1).

Monocytes comprised the main infected cell population *in vivo*, first detectable at DPI 4 and with an increasing frequency thereafter (Figure 2I). Consistent with previous studies, T cells, B cells, and neutrophils were not identified as infected more often than expected by chance or than MDCK control cells. We did not observe any infected plasmacytoid (pDCs) or conventional dendritic cells (cDCs) in circulation, though infected DCs have been observed in culture and in lymph nodes (Geisbert et al., 2003c).

Monocytes are avid phagocytes and their intracellular viral RNA could reflect internalization of infected cell debris rather than infection. However, neutrophils also phagocytose debris from infected cells (Hashimoto et al., 2007) but do not support productive EBOV replication (Mohamadzadeh et al., 2006). Indeed, we do not detect neutrophils to be infected more often than expected by chance (Figure 2I), consistent with rapid RNA degradation inside of phagosomes (Huang et al., 2015). Moreover, infected monocyte transcriptomes predominantly contained viral mRNA rather than genomic RNA from extracellular virions (see later Results). These observations suggest that most cells with viral RNA reflect viral transcription rather than uptake of infected cell debris or viral particles, though we cannot exclude the latter possibilities in every cell.

### Interferon Response Drives Gene-Expression Programs across Multiple Cell Types

Having examined the varying abundance of each immune cell type, we next cataloged changes in their gene-expression profiles throughout EVD. We grouped cells into EVD stages: “incubation,” preceding detectable viral load or clinical signs (DPI 1 and 2; CyTOF only); “early,” detectable viral load but no clinical signs (DPI 3); “middle,” both viral load and clinical signs (DPI 4 and 5); and “late,” when NHPs reached euthanasia criteria (DPI 6–8) (Figure 1).

**Figure 3:**
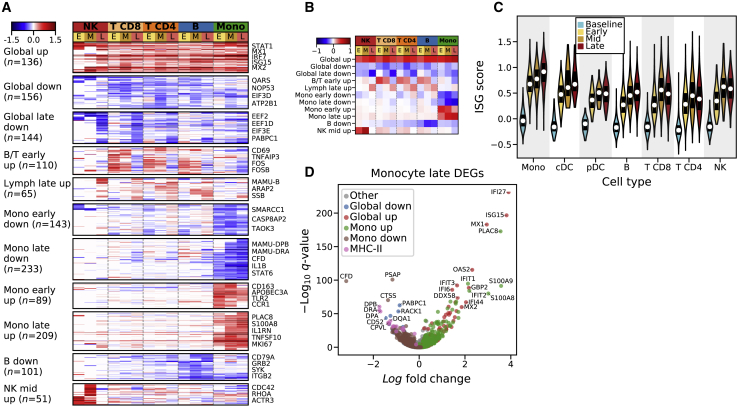
Patterns of Differential Expression across EVD Stages and Cell Types (A) Fold changes (log_*e*_ scale) of 1,430 differentially expressed genes (rows) in each cell type at early (E), middle (M), and late (L) EVD (columns), relative to baseline, with insignificant values (p > 0.2) set to 0. Genes were grouped into modules through unsupervised *k*-means clustering. See also Tables S2 and S3. (B) Same as (A) but displaying the average log_*e*_ fold change of each module. (C) Distribution of interferon-stimulated gene (ISG) scores for each cell type. White markers: median; bars: interquartile range. See also Figures S4A and S4B. (D) Differential expression of monocytes in late EVD compared to baseline.

We compared transcriptomes from each EVD stage to baseline for each cell type (STAR Methods) and identified 1,437 genes that were differentially expressed in at least one cell type and stage with a false discovery rate (FDR) corrected *q* value <0.05 and a fold change of >30% (Table S2). To identify patterns of gene expression associated with cell type and time, we performed unsupervised clustering of the differential expression signatures and identified 11 gene modules (Figures 3A and 3B; Table S3). We excluded neutrophils, pDCs, cDCs, and plasmablasts because of small sample sizes. Three modules that we term “global” were broadly up or downregulated compared to baseline across cell types, and the remaining modules were cell-type specific.

**Figure S4:**
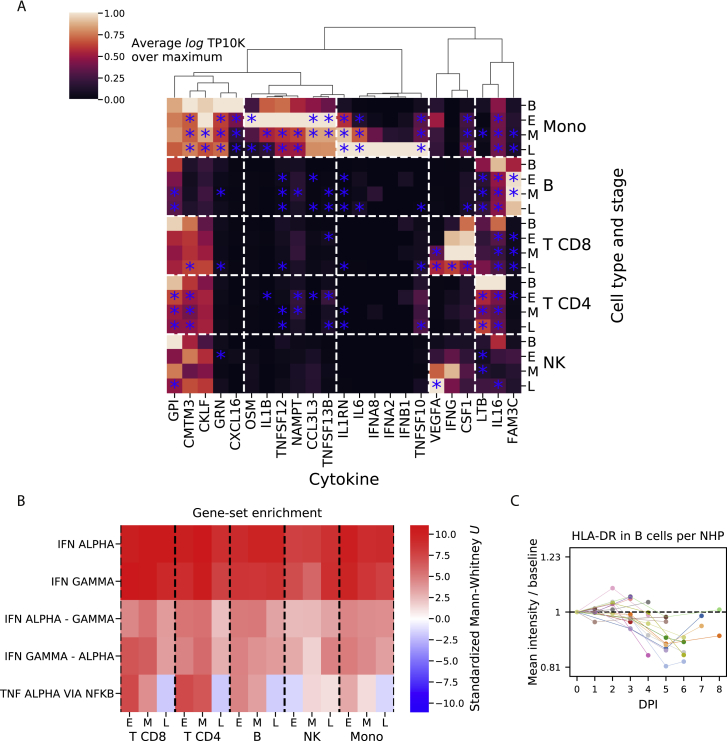
Quantification of Cytokine Expression and Enrichment of Response Signatures, Related to Figures 3 and 4 (**A**) Average expression values (log_*e*_ TP10K) of literature-annotated cytokines (columns) across cell types and stages of acute EVD (rows). Values are plotted as a ratio relative to the maximum across cell types and stages. Values that are statistically different from baseline (p < 0.05) are indicated with a blue star. (**B**) Heatmap of rank-sum test statistics for comparison of differential expression log fold-changes of genes in a gene set (rows) compared to genes not in the set. The log fold-changes were defined from differential expression profiles of each cell type at each EVD stage (columns) relative to baseline. Five gene sets were tested — three from the Hallmark database (IFN ALPHA, IFN GAMMA, and TNF ALPHA VIA NFKB) (Liberzon et al., 2015) and 2 constructed from the hallmark sets, as uniquely IFNα-regulated genes in “IFN ALPHA” but not “IFN GAMMA” (“IFN ALPHA - GAMMA”), and vice versa for uniquely IFNγ-regulated (“IFN GAMMA - ALPHA”). See also Table S3. (**C**) Fold change (log_2_ scale) in average HLA-DR CyTOF intensity on B cells at each DPI relative to baseline for each PBMC sample. Colored lines connect serial samples from the same NHP.

The “global up” module contained 136 genes, consisting mostly of regulators and targets of the interferon (IFN) alpha (α) and gamma (γ) signal transduction cascade such as *STAT1* and *MX1*. Gene sets labeled “response to IFN-α,” and “response to IFN-γ” were significantly enriched in this module (IFN-α: odds ratio [OR] = 69.5, *q* = 8 × 10^−39^; IFN-γ: OR = 45.9, *q* = 1 × 10^−39^; Fisher’s exact test; Table S3). This IFN response was consistent with a >10-fold increase of IFN-γ mRNA in CD8^+^ T cells from 0.4 transcripts per ten thousand (TP10K) at baseline to 5.0 at mid EVD (Figure S4A). Type 1 IFN (α/β) mRNAs also increased, but the change was not statistically significant, as IFN-α/β mRNAs are expressed transiently (Lin et al., 2011). To further characterize the IFN response, we scored each cell by the expression of literature-annotated ISGs. The median ISG score increased for all cell types and periods (p < 1 × 10^−5^, rank-sum test, Figure 3C).

To determine whether IFN-α, IFN-γ, or both were driving this response, we identified genes that were annotated as regulated by only one of the two. Both uniquely IFN-α- and uniquely IFN-γ-regulated genes were significantly enriched in the “global up” module (*q* < 0.01, IFN-α: OR = 20.9; IFN-γ: OR = 16.6), a pattern that held true for each cell type and EVD stage (Figure S4B). These results suggest that both IFN-α and IFN-γ substantially and independently influenced the gene-expression profiles of circulating cells during EVD.

The “global late down” module contained 144 genes that were predominantly downregulated during late EVD. It contained numerous regulators of translation (REACTOME_TRANSLATION gene set enrichment *q* = 5.2 × 10^−7^, Table S3), which is consistent with a core antiviral function of IFN being to downregulate translation (Li et al., 2015).

### Cell Type and Temporally Specific Modules Underlie Cell States

After elucidating the global effects of EBOV infection on immune cells, we next investigated the transcriptional responses specific to each cell type.

The 2 modules “B/T early up” and “lymph late up” reflect changing gene expression of lymphocytes at different stages of EVD. “B/T early up” is strongly associated with the gene set HALLMARK_TNFA_SIGNALING_VIA_NFKB (*q =* 1.3 × 10^−9^), including the canonical activation marker *CD69* (Testi et al., 1994), *CD48* (McArdel et al., 2016), and the transcription factor *FOS* (Foletta et al., 1998). This module is unlikely to represent antigen-dependent activation as it occurs in most lymphocytes, and several of the top upregulated genes (e.g., *GADD45B*, *ZFP36L2)* are associated with growth arrest rather than proliferation. In addition, the 5^th^ most enriched gene set in the “B down” module reflects BCR signaling (*q* = 0.00017), i.e., reduced BCR activation during EVD. Thus, the “B/T early up” module likely represents a cytokine-mediated, non-antigen-dependent activation of lymphocytes.

The “lymph late up” module is upregulated in late EVD across all lymphocytes. The top associated gene sets implicate DNA repair (*q* = 0.00031) and apoptosis via TRAIL (*q* = 0.00032). This latter gene set is consistent with previous reports of T cell apoptosis in EVD (Iampietro et al., 2017; Wauquier et al., 2010) and with the decline in lymphocyte abundance in our dataset (Figure 2D).

### Monocytes Express Reduced MHC Class II mRNAs and Proteins Independent of Infection Status

Monocytes were of particular interest as they are well known to be central to EVD pathogenesis (Geisbert et al., 2003b; Lüdtke et al., 2016). Consistent with previous observations (Menicucci et al., 2017), we found that monocytes had far more significant gene-expression changes than the other cell types: 1,020 genes (11.6% of genes tested) in at least one EVD stage versus baseline, compared to 505 genes (6.6%) for B cells, which ranked second. We therefore focused our attention on characterizing monocytes.

One prominent feature of the monocyte differential expression profile was the downregulation of several major histocompatibility complex (MHC) class II genes by mid and late EVD (Figure 3D). Monocytes and professional antigen-presenting cells display viral antigens on MHC class II proteins at the cell surface to stimulate the adaptive immune response. While IFN-γ typically upregulates MHC class II gene expression (Steimle et al., 1994), we observed decreased MHC class II on monocytes despite elevated *IFN-γ* mRNA levels in T cells (Figure S4A) and widespread IFN-γ transcriptional response in monocytes (Figure S4B). Previous reports have described loss of HLA-DR, one of the 4 MHC class II proteins, during EBOV infection of monocytes *ex vivo* (Hensley et al., 2002), and NHPs and humans *in vivo* (Menicucci et al., 2017; Lüdtke et al., 2016). However, the specific MHC genes affected, cell-type specificity, temporal dynamics, and relationship with EBOV infection status, have not been previously described.

**Figure 4:**
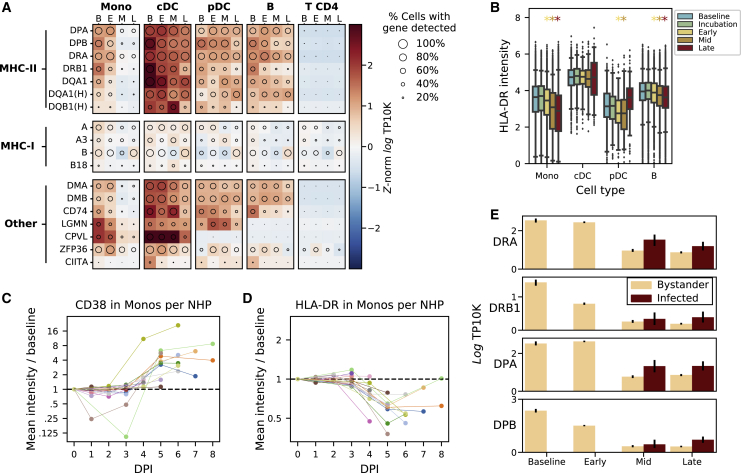
Monocytes Dramatically Reduce Expression of MHC Class II Proteins Independent of Infection Status (A) Expression of major histocompatibility (MHC) or MHC-associated genes (rows) in key cell types at baseline (B), early (E), middle (M), or late (L) EVD (columns). Circle size: percentage of cells in that group in which the gene was detected; color: mean expression in *Z* score normalized, log_*e*_ transcripts per 10,000 (TP10K). The “MAMU-” prefix, which designates MHC genes in rhesus monkeys, was removed; the “HLA-” prefix is indicated by “(H).” (B) CyTOF intensity of HLA-DR protein in antigen-presenting cells. Boxes: median and interquartile range; whiskers: 2.5^th^ and 97.5^th^ percentiles. Colored stars indicate significant decreases from baseline (rank-sum test p < 0.05) with color corresponding to stage. (C and D) Fold change (log_2_ scale) in average CD38 (C) and HLA-DR (D) CyTOF intensity on monocytes at each DPI relative to baseline, connected by colored lines for each NHP. See also Figure S4C and Data S1. (E) Average gene expression (log_*e*_ TP10K) for four MHC class II genes in monocytes, stratified by cell-infection status. Error bars: 95% CI on the mean based on 200 bootstraps.

We observed widespread changes in MHC expression throughout EVD (Figure 4A). The most striking decreases occurred in MHC class II genes of monocytes (>5-fold for *DPA*, *DPB*, and *DRA* by late EVD, all *q* < 1 × 10^−21^), with smaller changes in MHC class I genes (<1.7-fold increase for *A*, *A3*, and *B*, *q* < 5 × 10^−4^). B cells displayed modest reductions in MHC class II genes as well (>1.9-fold for *DPA*, *DPB*, *DRA*, and *DQA1*, *q* < 1 × 10^−22^). pDCs and cDCs showed no statistically significant reduction of any MHC class II gene (*q* > 0.05), but our dataset contained few DCs (Table S1) so we had less power to detect these effects. CyTOF revealed similar patterns in protein levels (p < 1 × 10^−61^ for monocytes at all stages, rank-sum test; p = 0.0012 for B cells at late EVD, Figures 4B and S4C). This phenomenon held true for each individual NHP: even as monocytes became activated, demonstrated by upregulation of the canonical activation marker CD38 (Amici et al., 2018) (Figure 4C; Data S1), they showed dramatic downregulation of HLA-DR protein expression at DPI 5–8 (p = 9.5 × 10^–7,^ Figure 4D).

Reduced MHC class II expression in monocytes was not a direct consequence of EBOV infection of those cells. Only a small (∼5%) percentage of monocytes were infected at mid EVD (Figure 2I), too small to explain the size of the reduction. In fact, expression of MHC class II genes was comparable or higher in infected cells than in uninfected (bystander) cells (Figure 4E).

We identified genes correlated with MHC class II in monocytes (STAR Methods), as they may be part of a co-regulated expression program. Many of the most correlated genes are functionally involved in antigen presentation, including *CD74*, *LGMN*, and *B2M* (Figure 4A; Spearman ρ = 0.42, 0.41, 0.33, respectively, all p < 10^−170^). One of the most associated genes was *ZFP36* (Spearman ρ = 0.43, p < 10^−296^), which directly regulates turnover of MHC class II and other immune-related mRNAs (Pisapia et al., 2019). Thus, MHC class II and other genes involved in antigen presentation may be part of a single transcriptional module, co-regulated by *ZFP36* and/or other genes.

### Differentially Expressed Genes between Infected and Bystander Monocytes

**Figure 5:**
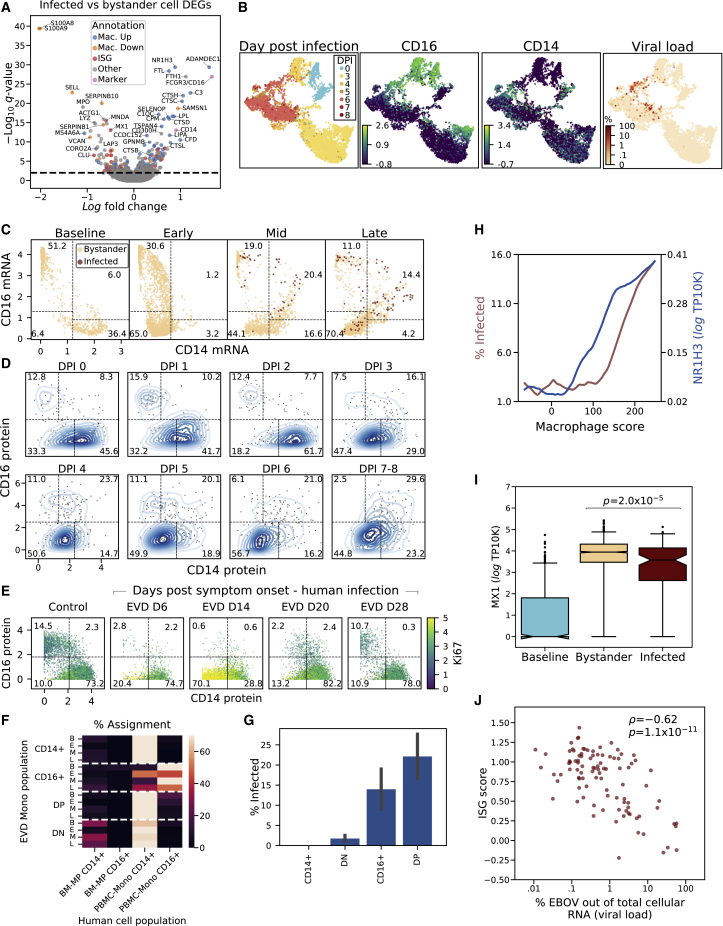
ISG Suppression, Co-expression of CD14 and CD16, and Expression of Macrophage Genes Are Associated with Monocyte Infectivity (A) Differential expression between infected and bystander monocytes from DPI 5–8. Genes are colored by membership in sets of genes (Mac. Up/Down = up- or downregulated during *in vitro* differentiation of monocytes into macrophages). See also Table S4. (B) UMAP embedding of monocyte gene expression data, colored by (left-to-right) DPI, *CD16* expression (log_*e*_ TP10K), *CD14* expression (log_*e*_ TP10K), and percentage of cellular transcripts mapping to EBOV. (C) Smoothed expression (log_*e*_ TP10K) of *CD14* and *CD16* for monocytes during EVD. Boxes: CD14^+^, CD16^+^, DN, and DP subsets described in the text; numbers: percentage of cells in each subset at that EVD stage. See also Figures S5A and S5B. (D) CD14 and CD16 protein expression (CyTOF intensity) on monocytes at each DPI. Bivariate kernel density plot with 200 randomly sampled cells is overlaid as a scatterplot. See also Figure S5C. (E) CD14 and CD16 protein expression (CyTOF intensity) on monocytes in a case of human EVD, colored by Ki67 protein expression for multiple days after symptom onset. See also Figure S5D. (F) Percentage of assignment of NHP CD14/CD16 subsets at each EVD stage to human myeloid reference populations (BM-MP: bone marrow monocyte progenitors, PBMC-CD16^+^: circulating CD16^+^ monocytes, PBMC-CD14^+^: circulating CD14^+^ monocytes). See also Figures S5E–S5K. (G) Percentage of infected monocytes in each CD14/CD16 subset in late EVD. Error bars: 95% CI on the mean based on 1,000 bootstraps. (H) Association between macrophage score (x axis) and percentage of infected cells (left y axis, red) and expression of the differentiation marker *NR1H3* (right y axis, blue, log_*e*_ TP10K). We ordered monocytes from late EVD by macrophage score, and averaged percentage of infected cells and *NR1H3* expression within 400-cell sliding windows. See also Figures S6A–S6C. (I) *MX1* expression (log_*e*_ TP10K) in monocytes at baseline, and uninfected bystanders or infected cells in late infection. Boxes: median and interquartile range; whiskers: 2.5^th^ and 97.5^th^ percentiles. Statistical significance was assessed by rank-sum test. See also Figure S6D. (J) Scatterplot of ISG score (y axis) versus percentage of cellular transcripts mapping to EBOV (x axis) for infected monocytes in late EVD (DPI 6–8). Statistical significance was assessed by Spearman ρ.

Next, we characterized genes that were differentially expressed between infected and bystander monocytes, as these could represent host entry factors, restriction factors, or genes that are regulated by infection within a cell. For this and subsequent analyses, EBOV transcripts were excluded from library size normalization to avoid bias in estimating host gene expression in infected cells (STAR Methods). 505 genes were differentially expressed between infected and bystander monocytes (*q* < 0.05) of which 276 changed by >30% (Figure 5A; Table S4).

We observed 3 broad categories of differentially expressed genes: those associated with monocyte subtypes, monocyte-to-macrophage differentiation, and ISGs.

### Emergence of CD14^–^ CD16^–^ Immature Monocyte Precursors Suggests Emergency Myelopoiesis

*CD14* and *FCGR3* (which codes for CD16) were overexpressed in infected monocytes relative to bystanders. These two genes define classical (CD14^+^) and non-classical (CD16^+^) monocytes (Kapellos et al., 2019), which are the dominant blood monocyte subsets in healthy individuals. Classical monocytes are highly phagocytic scavenger cells, while non-classical monocytes are involved in complement and antibody-mediated phagocytosis. At baseline, these subsets were easily detectable as 2 distinct clusters, marked by high expression of *CD14* (Figure 5B, bottom half of the blue lobe of DPI panel) or *CD16* (top half of the blue lobe).

However, monocyte subsets changed dramatically during EVD. Both single-positive CD14^+^ and CD16^+^ monocytes declined, while 2 unusual populations emerged: a large population of CD14^–^ CD16^–^ cells (double negatives [DNs]) and a smaller population of CD14^+^ CD16^+^ cells (double positives [DPs]) (Figure 5C). While 87.6% of cells fell into single CD14^+^ or CD16^+^ bins at baseline, this dropped to 33.8%, 35.6%, and 15.2% in early, middle, and late EVD, respectively. We confirmed this pattern at the protein level by CyTOF (Figure 5D).

**Figure S5:**
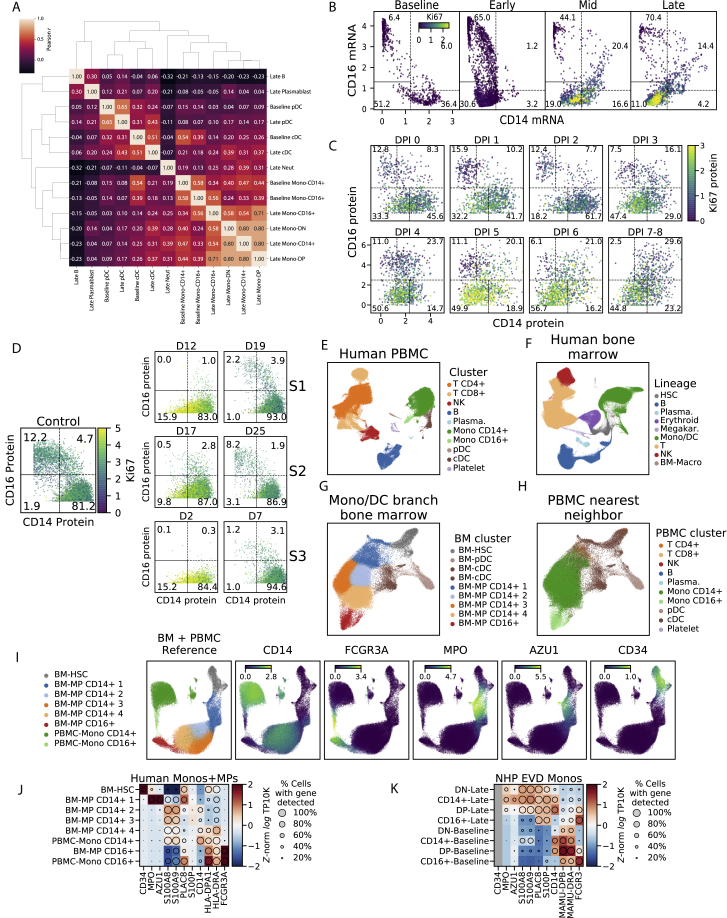
Extended Characterization of Interferon and Double-Negative CD14^–^ CD16^–^ Monocytes, Related to Figure 5 (**A**) Clustermap of pairwise Pearson correlations between cell type clusters at baseline and late EVD. Correlations are computed on average log_*e*_ TP10K expression values of overdispersed genes. DN and DP monocytes at late EVD are more similar to monocytes (including baseline CD14+s) than other cell types. (**B**) Scatterplot of MAGIC-smoothed expression values (log_*e*_ TP10K) of *CD14* and *CD16* for monocytes in baseline, early, mid, and late disease stages. Cells are colored by smoothed expression levels of *MKI67* (the gene coding for Ki67 protein). Boxes: CD14+, CD16+, DN, and DP subsets described in the text; numbers: percentage of cells falling into each subset. (**C**) Scatterplot of protein expression (CyTOF intensity) of CD14 and CD16 for 1,000 randomly sampled monocytes at each DPI. Cells are colored by Ki67 expression. Boxes: CD14+, CD16+, DN, and DP subsets described in the text; numbers: percentage of cells falling into each subset. (**D**) Scatterplot of protein expression (CyTOF intensity) of CD14 and CD16 for monocytes during human EVD. Left: monocytes from healthy human controls. Right: monocytes from 3 EVD cases (S1, S2, and S3) at various days post symptom onset. Cells are colored by Ki67 marker intensity. Boxes: CD14+, CD16+, DN, and DP subsets described in the text; numbers: percentage of cells falling into each subset. (**E**) UMAP embedding of healthy human PBMCs dataset, colored by annotated cluster assignment, based on known marker genes. (Plasma.: Plasmablast). (**F**) UMAP embedding of healthy bone marrow cells, colored by cluster assignment, based on marker genes. (HSC: hematopoietic stem cell, Plasma.: Plasmablast, Megakar.: Megakaryocyte, Mono/DC: monocyte and dendritic cell, BM-Macro: bone marrow macrophage). (**G**) UMAP embedding of sub-clustered HSC and monocyte/dendritic lineage cells. (BM: bone marrow, MP: monocyte progenitor) (**H**) Same UMAP embedding as Figure S5G, but colored by the cluster identity of their nearest neighbor in the human PBMC dataset (Figure S5E). (**I**) UMAP embedding of the merged reference dataset of healthy bone marrow HSCs and monocyte lineage cells and PBMCs. Left sub-panel is colored by cluster assignment. Right sub-panels are colored by marker gene expression (log_*e*_ TP10K). (**J**) Expression profiles of selected genes for human bone marrow monocyte progenitors (BM-MPs) and human circulating monocytes (PBMC-Monos). Circle area: percentage of cells in which the gene was detected; color: average expression (*Z*-normalized log_*e*_ TP10K). (**K**) Expression profiles of selected genes for NHP monocyte subsets at baseline or late EVD for orthologs of the genes in (J). Circle area: percentage of cells in which the gene was detected; color: average expression level (*Z*-normalized log_*e*_ TP10K). *CD34* is grayed out because it is detected in <10 cells.

At late EVD, the most frequent population was DN cells, which rose to make up 70.4% (by scRNA-seq) or 56.7% (by CyTOF) of monocytes. As they expressed neither CD14 nor CD16, we confirmed that their gene-expression profiles were most correlated with conventional monocytes and not other cell types (Figure S5A). DNs first emerged on DPI 3, coinciding with the 2-fold increase in monocytes (Figure 2D), and with the increased monocyte proliferation observed in mid EVD (Figure 2G). The DN population is in fact highly proliferative: while 0% of monocytes at baseline expressed elevated levels of Ki67 (smoothed log TP10K >1), 37% of DN monocytes exceeded this threshold by late EVD (Figures S5B and S5C). Therefore, DNs underlie the increased monocyte proliferation in mid EVD.

To determine whether DNs arise in human EVD, we re-analyzed published CyTOF data from 4 patients during the 2013–2015 epidemic (McElroy et al., 2020) (STAR Methods). All of the human cases showed a loss of conventional CD14^+^ and CD16^+^ single-positive monocytes and an emergence of proliferative DN monocytes during acute disease (Figures 5E and S5D). Then, the DNs disappeared and were replaced by conventional CD14^+^ and CD16^+^ monocytes as the patients recovered. Thus, DNs are a feature of human sub-lethal cases and our lethal NHP model.

Mature monocytes in circulation are non-dividing (van Furth et al., 1979), but infection and cancer can induce the release of proliferating immature myeloid cells from the bone marrow, a process known as emergency myelopoiesis (Chiba et al., 2018; Cuenca et al., 2015; Al Sayed et al., 2019). We therefore hypothesized that the DN population may derive from emergency myelopoiesis.

If DNs result from emergency myelopoiesis, their gene expression may be more similar to bone marrow monocyte precursors (BM-MPs) than circulating monocytes. To test this, we analyzed a reference scRNA-seq dataset of BM-MPs from healthy human bone marrow (Hay et al., 2018) and mature monocytes from human PBMCs (Figures S5E–S5H; STAR Methods). Compared to mature monocytes, BM-MPs showed lower expression of *CD14* and *FCGR3A* (the human *CD16* gene) and higher expression of *MPO*, *AZU1*, *S100A8*, and *S100A9* (Figures S5I and S5J). In our data, the expression of those genes in DNs at late EVD relative to baseline monocytes mirrored BM-MPs, suggesting their similarity (Figure S5K). Moreover, in a nearest-neighbor test (STAR Methods), DNs were more likely, than conventional subtypes, to be matched with BM-MPs (Figure 5F). Thus, DNs may represent immature monocytes released from the bone marrow in response to the EVD cytokine milieu.

### Monocytes Expressing Markers of Macrophage Differentiation Are Enriched for EBOV Infection

In addition to DNs, we also observed CD14^+^ CD16^+^ DP cells, which rose to make up 20.4% of the monocytes by mid EVD (Figure 5C). A similar increase in DP monocytes has been observed in other viral infections (Michlmayr et al., 2018; Zanini et al., 2018a). We found that the DP population harbored a disproportionately high percentage of EBOV-infected cells (Figure 5G), consistent with the fact that *CD14* and *CD16* were each higher in EBOV-infected monocytes than in bystanders (Figure 5A). At late EVD, 22.1% of DPs were infected compared to only 1.7% of DNs. Thus, the differential expression of *CD14* and *CD16* in infected cells results from increased infection of the DP cells, rather than increased expression of *CD14* on classical and *CD16* on non-classical monocytes.

**Figure S6:**
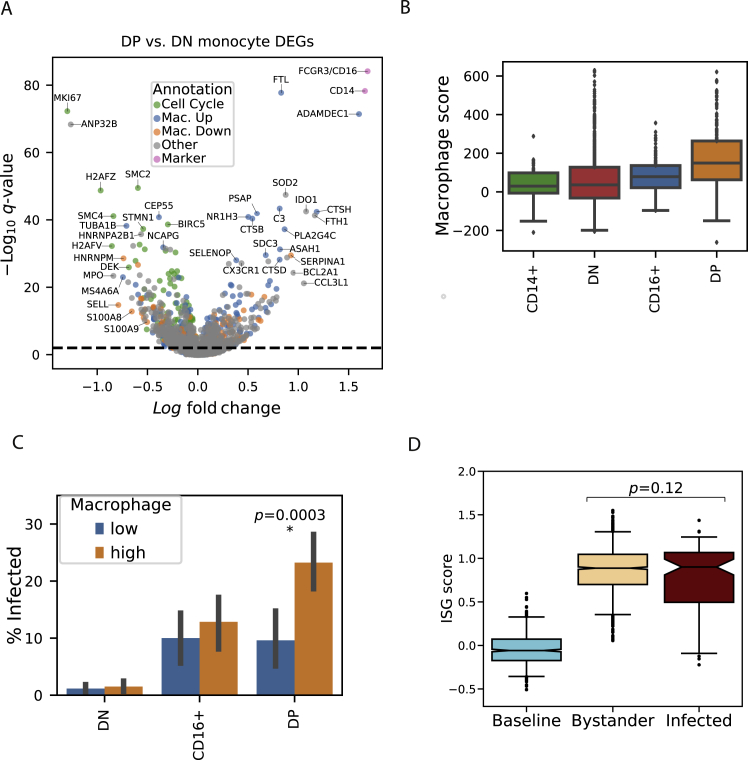
Extended Characterization of Gene-Expression Signals Associated with EBOV Infection Status in Monocytes, Related to Figure 5 (**A**) Volcano plot of differentially expressed genes between double positive and double negative monocyte subsets from DPI 5–8. Genes are colored by membership in cell cycle, macrophage upregulated (Mac. Up), macrophage downregulated (Mac. Down), or marker (*CD14*, *CD16*) gene sets. See also Table S5. (**B**) Macrophage scores for monocytes in late EVD for each subset. Boxes: median and interquartile range; whiskers: 2.5th and 97.5th percentiles. (**C**) Percentage of infected monocytes in each subset in late disease, stratified by low or high macrophage score (below or above the median of monocytes from all subsets). Error bars: 95% bootstrap CI on the mean. Statistical significance was assessed by Fisher's exact test. There are no infected monocytes in the CD14+ subset. (**D**) ISG scores of monocytes at baseline, and uninfected bystanders or infected cells in late stage EVD (DPI 6–8). Boxes: median and interquartile range; whiskers: 2.5th and 97.5th percentiles. Statistical significance was assessed by rank-sum test.

Differentially expressed genes between infected and bystander monocytes (Figure 5A; Table S4) *and* between DP and DN monocytes (Figure S6A; Table S5) were enriched for monocyte-to-macrophage differentiation associated genes, including known EBOV entry factors. Freshly isolated monocytes are largely refractory to EBOV infection in cell culture, but EBOV entry factors are upregulated during *in vitro* macrophage differentiation, allowing increased infection (Martinez et al., 2013). *In vivo*, we observed higher levels of macrophage differentiation markers in infected cells than in bystanders, including the known EBOV entry factors cathepsin L (*CTSL*) and B (*CTSB*), and *GNPTAB* (Carette et al., 2011; Gnirss et al., 2012) (*q* = 6.7 × 10^−9^, 3.8 × 10^−7^, and 2.1 × 10^−3^, respectively). By contrast, the cellular receptor *NPC1* was not significantly differentially expressed, suggesting that natural variability in *NPC1* mRNA does not influence EBOV infectivity within circulating monocytes *in vivo*.

To determine whether upregulation of these factors was part of a general macrophage differentiation program, we used gene sets derived from published bulk RNA-seq data of primary blood monocytes before and after differentiation into macrophages *in vitro* (Dong et al., 2013). Genes that are upregulated during *in vitro* differentiation were significantly enriched in infected cells (OR = 3.5, p = 3.1 × 10^−11^, Fisher’s exact test), while downregulated genes were significantly enriched in bystanders (OR = 3.7, p = 4.2 × 10^−8^, Fisher’s exact test; combined chi-square goodness of fit test p = 2.2 × 10^−30^). Additional differentiation studies yielded similar findings (chi-square goodness of fit test p = 2.6 × 10^−9^ [Saeed et al., 2014], Fisher’s exact test p = 5.7 × 10^−12^ [Italiani et al., 2014]). Genes associated with differentiation into M2-polarized macrophages were more enriched among infected cells than those of M1-polarized macrophages (OR = 7.8, p = 1.3 × 10^−10^ versus OR = 3.3, p = 1.6 × 10^−3^ (Italiani et al., 2014), Table S4).

The proportion of infected cells increased along with a score reflecting the expression of genes associated with macrophage differentiation (STAR Methods). Over the range of scores, we observed that the percentage of infected cells rose ∼5-fold from 3% to 15% (Figure 5H).

Among the monocyte subsets, DPs generally had the highest macrophage scores (Figures S6A and S6B). Thus, some of the enrichment of infected cells among the DP subset could be attributed to their more macrophage-like gene expression.

However, there was substantial heterogeneity in macrophage scores within monocyte subsets, and macrophage score and CD14/CD16 subset were independently predictive of infectivity. Stratifying cells in each subset by macrophage score, less macrophage-like DPs were still more likely to be infected than more macrophage-like DNs (p = 1.9 × 10^−25^, Fisher’s exact test, Figure S6C). Yet, within the DPs, more macrophage-like cells were more likely to be infected than less macrophage-like cells (p = 0.0003, Fisher’s exact test, Figure S6C). Similarly, when we fit a logistic regression predicting the infection status of each cell using macrophage score, smoothed *CD14* and *CD16* expression values, and a *CD14*x*CD16* interaction term (STAR Methods), both the *CD14*x*CD16* interaction term (which is highest in DPs) and macrophage score were positively associated with infection, while the *CD14* and *CD16* terms were negatively associated (p < 0.01 for all coefficients).

### ISGs Are Downregulated in Infected Monocytes Relative to Bystanders

Finally, we noticed that several key ISGs such as *MX1* were expressed at lower levels in infected cells than in bystanders (MAST *q* = 7.7 × 10^−14^, rank-sum test p = 2.0 × 10^−5^, Figures 5A and 5I). To determine whether infection suppressed overall ISG expression, we assessed the magnitude of the IFN response between infected and bystander cells at late EVD. While both bystander and infected monocytes at late EVD had higher ISG scores than monocytes at baseline, ISG scores were lower in infected cells than bystanders (not statistically significant by rank-sum test, Figure S6D). More strikingly, there was a significant negative correlation between ISG score and the percentage of cellular transcripts derived from EBOV (i.e., the intracellular viral load) (Spearman ρ = −0.62, p = 1.1 × 10^−11^, Figure 5J). This suggests that ISGs are downregulated during viral replication within infected cells (see later Results).

### Single-Cell Transcriptomics of *Ex Vivo*-Infected PBMCs Reveals Temporal Dynamics in Viral Gene Expression

**Figure 6:**
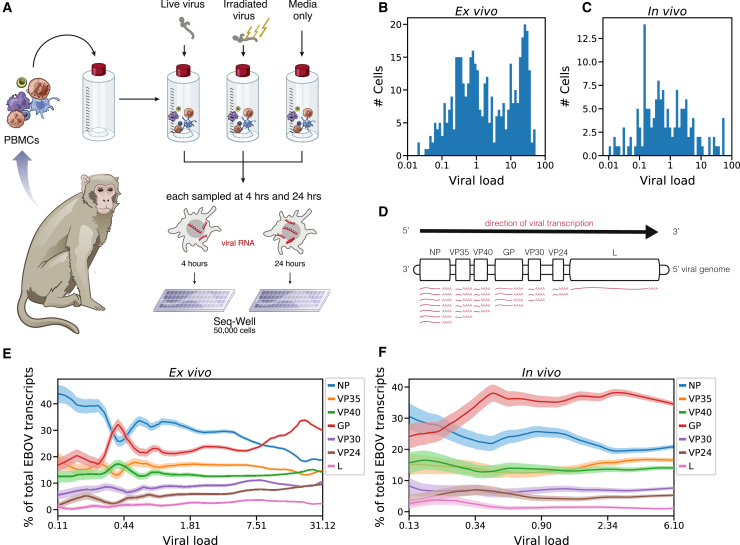
Viral Transcriptional Dynamics of Infected Monocytes *In Vivo* and *Ex Vivo* (A) Schematic of EBOV challenge of PBMCs *ex vivo*. See also Figure S7. (B and C) Percentage of cellular transcripts derived from EBOV (intracellular viral load) in monocytes from PBMCs inoculated with live virus *ex vivo* (B) or from PBMCs of NHPs infected *in vivo* (C). See also Figures S8A–S8D. (D) Schematic of EBOV transcription. The viral RNA-directed RNA-polymerase transcribes each gene sequentially but occasionally releases the genomic RNA template, ending transcription. As a result, transcription frequency decreases from NP to *L*. (E and F) Proportion of each EBOV gene versus viral load (log_10_ scale), *ex vivo* (E) or *in vivo* (F). We ordered infected monocytes by viral load and averaged the percentage of each viral gene over 50-cell sliding windows. Bands: mean ± 1 SD. See also Figures S8E and S8F.

In order to more thoroughly probe viral and host gene expression during the viral life cycle, we isolated PBMCs from 2 healthy rhesus monkeys (NHP1 and NHP2) and inoculated them *ex vivo* with live EBOV, EBOV rendered replication-incompetent by gamma irradiation (Feldmann et al., 2019), or only media as a control (Figure 6A). We performed scRNA-seq using Seq-Well at 4 h or 24 h post-infection (HPI), corresponding to early (start of viral transcription) and middle-to-late stages (viral genome replication, virion assembly) of the viral life cycle. Unlike alternative systems such as virion-like particles, inoculation with gamma-irradiated EBOV allowed us to profile cells containing viral RNA and characterize the host response in the absence of effective viral transcription and translation.

**Figure S7:**
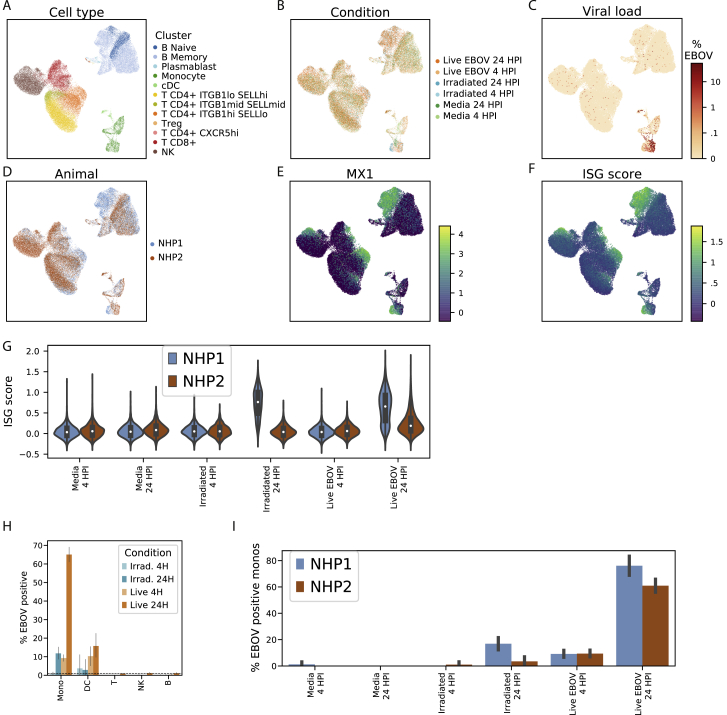
Overview of the *Ex Vivo* EBOV Infection Dataset, Related to Figure 6 (**A–F**) UMAP embedding of Seq-Well data colored by annotated cluster assignment (A), treatment condition (B), viral load (C), NHP donor (D), *MX1* gene expression (log_*e*_ TP10K) (E), and interferon stimulated gene (ISG) score (F). (**G**) Distributions of ISG scores across monocytes from each treatment condition, stratified by NHP donor. Central white marker: median; black bar: interquartile range. (**H**) Estimated percentage of infected cells of each cell type in the *ex vivo* dataset. The dashed line denotes the 1% false positive rate threshold used for calling infected cells. Error bars: 95% bootstrap CI on the mean. (**I**) Percentage of EBOV-positive monocytes from each *ex vivo* treatment condition, stratified by NHP donor. Error bars: 95% bootstrap CI on the mean.

We obtained single-cell transcriptomes from 50,646 PBMCs *ex vivo* and observed similar cell-type representation, clustering by treatment condition, and distribution of EBOV-infected cells as with the *in vivo* collections (Figures S7A–S7C). ISG scores were higher in NHP1 than NHP2 (Figures S7D–S7G), so we analyzed cells from each NHP separately and jointly to avoid potential artifacts. While we did not observe infected DCs *in vivo*, we found that 16% of DCs (16.0% in NHP1, 15.7% in NHP2) inoculated with live virus *ex vivo* were infected by 24 HPI (Figure S7H).

**Figure S8:**
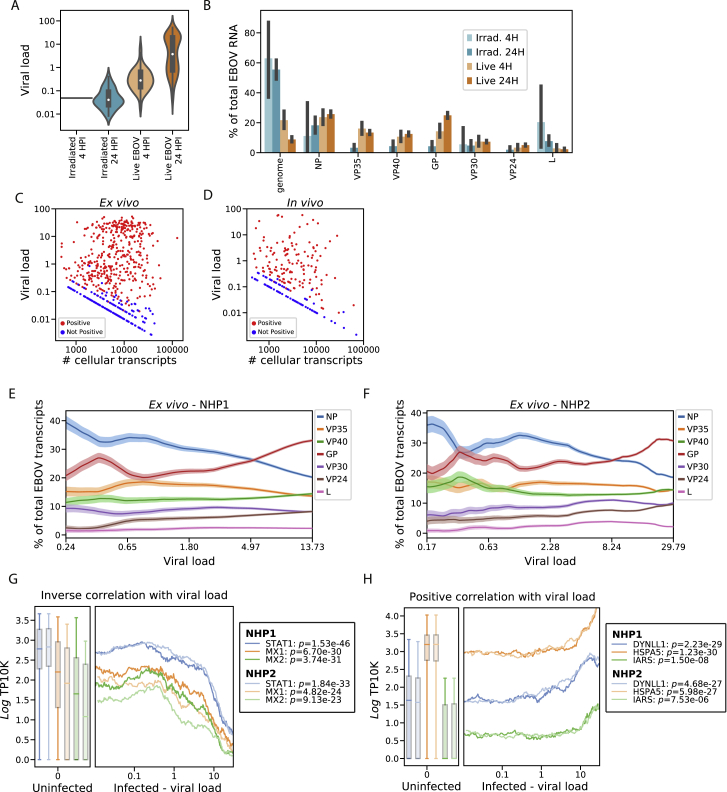
EBOV Infection Dynamics in the *Ex Vivo* Dataset, Related to Figures 6 and 7 (**A**) Distributions of viral loads across monocytes from different treatment conditions. Central white marker: median; black bar: interquartile range. (**B**) Estimated percentage of EBOV transcripts derived from the EBOV genome or each EBOV gene, out of total viral RNA, stratified by treatment conditions. Prior to averaging, the counts of EBOV genes for each cell was normalized to sum to one, so each cell contributes uniformly to the proportion, regardless of its total number of EBOV transcripts. Error bars: 95% bootstrap CI on the mean. (**C and D**) Scatterplot of total transcripts (unique molecular identifiers) detected in a cell (x axis, log_10_ scale) against viral load (y axis, log_10_ scale) for cells with one or more viral reads *ex vivo* (C) or *in vivo* (D). Cells called as infected are colored in red and otherwise colored in blue. (**E and F**) Relative proportion of each EBOV gene versus viral load (log_10_ scale) *ex vivo* for cells from donor NHP1 (E) or NHP2 (F). We ordered monocytes by viral load and averaged the percentage of each viral gene over 50-cell sliding windows. Color bands: mean ± 1 SD. (**G and H**) Association between gene expression and viral load for selected negatively (G) and positively (G) associated host genes in monocytes, 24 HPI after inoculation with live virus *ex vivo*. In the left sub-plots, distributions of gene expression in uninfected bystander cells are shown as a boxplot, boxes: interquartile range, whiskers: 2.5th and 97.5th percentiles. In the right sub-plots, we ordered infected cells by viral load (log_10_ scale) and averaged gene expression (log_*e*_ TP10K) over 100-cell sliding windows. Curves and box-plots are shown separately for the 2 donor NHPs. *p*-values for the Spearman correlation between viral load and gene expression are listed for each NHP donor in the legend.

*Ex vivo*, monocytes were the predominant infected cell type, with >65% infected by 24 HPI (Figures S7H and S7I). 11.8% of the monocytes treated with irradiated virus also contained a statistically significant number of viral reads, even though gamma irradiation damages the viral genome and eliminates productive replication (Feldmann et al., 2019). As expected, these cells had a significantly lower fraction of EBOV reads per cell than those treated with live virus (Figure S8A). Moreover, viral RNAs from the cells treated with irradiated virus were substantially less likely to be coding-sense mRNA transcripts than those from cells with live virus (44% versus 92%, Figure S8B). Thus, our method can detect fragments of viral genomic RNA from irradiated virus in cells, but these are not productive infections.

The intracellular viral load varies over several orders of magnitude (Figures 6B and 6C). While most cells harbored viral loads <0.1%, a substantial minority had loads >10%, with a maximum of 57.5% *in vivo* and 52.3% *ex vivo*. The observed heterogeneity was not due to differential transcriptome coverage, because cells with low and high viral load had a similar range of transcripts detected (Figures S8C and S8D).

The *ex vivo* data gave us an opportunity to test predictions based on established models of EBOV transcription. Transcription of EBOV’s 7 genes by the viral RNA-directed RNA polymerase L follows the canonical stop-start mechanism of filoviruses and other non-segmented negative-strand (NNS) RNA viruses (Brauburger et al., 2014, 2015). L initiates transcription at the 3′ end of the genome, and processes from 5′ to 3′; at each gene’s transcription termination signal, L either falls off the genomic RNA template or reinitiates transcription of the subsequent gene (Figure 6D; Mühlberger, 2007). *NP* is transcribed first and at the highest level, proceeding down the genome to *L* last and at the lowest level.

When we quantified the relative expression of EBOV genes as a function of viral load, we observed an unexpected accumulation of *GP* mRNA (Figures 6E and 6F) in both NHP1 and NHP2 (Figures S8E and S8F). At low viral loads, both *in vivo* and *ex vivo*, the gene-expression distribution roughly matched the expected pattern, with most transcripts derived from *NP*, and the fewest from the 5′ genes *VP30*, *VP24*, and *L*. In agreement with this pattern, cells inoculated with irradiated virus were highly enriched in *NP* mRNA (Figure S8B), suggestive of RNA fragment transcription. However, as viral load increased in cells infected with live EBOV, *GP* unexpectedly became the most abundant viral transcript. While higher levels of *GP* than *NP* RNA have been observed in previous bulk RNA-seq datasets (Menicucci et al., 2017; Bosworth et al., 2017; Albariño et al., 2018), our data indicate that this phenomenon occurs within individual cells, as a life-cycle-dependent regulation of viral gene expression.

### EBOV Infection Downregulates Host Antiviral Genes and Upregulates Putative Pro-viral Genes

**Figure 7:**
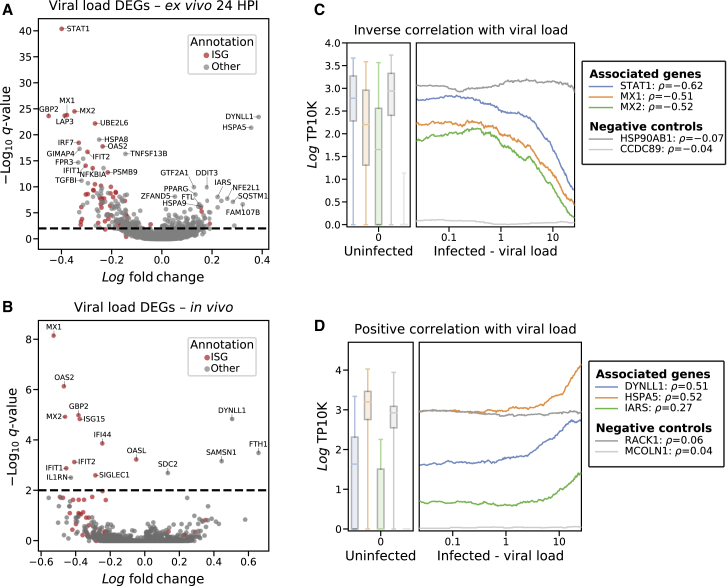
EBOV Infection Downregulates Host Antiviral Genes and Upregulates Putative Pro-viral Genes (A and B) Association between host gene expression and viral load within infected monocytes from PBMCs 24 HPI treated with live virus *ex vivo* (A) or from PBMCs of NHPs *in vivo* on DPI 5–8 (B). See also Table S6. (C and D) Select negatively (C) and positively (D) associated genes in monocytes from *ex vivo* infections. We ordered infected cells by viral load and averaged gene expression (log_*e*_ TP10K) over 100-cell sliding windows; Spearman correlation (ρ) is given in the legend. Boxplots show gene expression in uninfected cells (boxes: median and interquartile range; whiskers: 2.5^th^ and 97.5^th^ percentiles). See also Figures S8G and S8H.

Next, we exploited natural variability in viral load to identify host gene-expression changes correlated with viral replication, which may represent pathways directly regulated by infection. Instead of testing for differential expression between infected and bystander cells as we did previously, we looked for continuous association between viral abundance and host transcript levels in infected monocytes (STAR Methods). This identified 264 genes that were negatively correlated and 211 genes that were positively correlated with viral load *ex vivo* (*q* < 0.05), of which 34 changed by >30% per 10-fold increase in viral load (Figure 7A; Table S6).

Consistent with our previous observation that ISG score decreased with intracellular viral load, individual ISGs decreased dramatically with EBOV levels, both ex *vivo* and in *vivo* (e.g., *MX1*, *q* = 1.5 × 10^−24^, 7.4 × 10^–9^, respectively, Figures 7A and 7B). *Ex vivo*, the most negatively associated gene was *STAT1*, the master transcriptional regulator of the IFN response (*q* = 4.9 × 10^−41^ *ex vivo*, p = 0.0072 *in vivo* but not significant by FDR). Previous experiments have shown that the EBOV protein VP24 inhibits STAT1 activity by blocking its translocation to the nucleus (Reid et al., 2006). However, this is the first observation that *STAT1* mRNA levels decrease with viral replication within infected cells *in vivo*.

The expression of STAT1 and other negatively regulated ISGs remained relatively constant as EBOV levels rose to 1% of cellular transcripts but declined precipitously with higher EBOV levels (Figure 7C). This suggests that there is a delay before EBOV can downregulate host antiviral genes since it must transcribe and translate VP24 and other immunomodulatory proteins before they can act. The trajectories of these host antiviral genes were consistent between donor NHPs *ex vivo* (Figure S8G), even though more cells from NHP1 mounted an IFN response than NHP2.

Only a handful of host genes increased in expression alongside viral load, but their trajectories were consistent between the two NHPs (Figure S8H). The most dramatically upregulated gene was *DYNLL1* both *ex vivo* and *in vivo* (*q* = 2.5 × 10^−27^, 1.5 × 10^–5^, respectively, Figures 7A and 7B). DYNLL1 is a multi-functional protein involved in intracellular transport (Barbar, 2008). Intriguingly, DYNLL1 was previously shown to increase EBOV replication in a minigenome reporter assay (Luthra et al., 2015). Our data show that *DYNLL1* mRNA is upregulated, starting when EBOV RNA constitutes 0.1%–1% of transcripts, before ISGs decrease (Figure 7D).

Several other genes that we identified as upregulated alongside viral replication have known or speculated pro-viral functions in protein folding and synthesis. For example, *HSPA5* (*q* = 4.5 × 10^−22^ *ex vivo*, p = 0.04 *in vivo* but not significant by FDR) encodes a chaperone protein that is an essential host factor for EBOV (Reid et al., 2014), but upregulation of its mRNA has not been previously observed. Other hits such as *DDIT3* and *NFE2L1* (*q* = 1.3 × 10^–10^, 1.8 ×10^−8^ *ex vivo*, respectively) are sensors of ER and oxidative stress (Kim et al., 2016) and have been implicated in cell lines infected with Marburg virus (Hölzer et al., 2016) and in monocytes in EBOV-infected NHPs (Menicucci et al., 2017). We observe a corresponding enrichment of 3 gene sets associated with ER stress response, most notably BUYTAERT_PHOTODYNAMIC_THERAPY_STRESS_UP (*q* = 4.7 × 10^−14^, Fisher’s exact test, Table S6). Increased translation due to viral replication can drive cell stress and also deplete tRNAs and free amino acids (Albers and Czech, 2016). Upregulation of *IARS*—isoleucine tRNA synthetase (*q* = 8.4 × 10^−9^ *ex vivo*)—may reflect this cellular burden. 3 gene sets associated with depletion of amino acids were significantly upregulated, most notably KRIGE_RESPONSE_TO_TOSEDOSTAT (aminopeptidase inhibitor)_24HR_UP (*q* = 2.3 × 10^−6^, Fisher’s exact test, Table S6), suggesting that viral replication exhausts cellular amino acid stores. This hypothesis is consistent with prior observations of depleted amino acids in the plasma of fatal human EVD cases (Eisfeld et al., 2017).

## Discussion

Despite recurrent outbreaks, the molecular basis of EVD pathogenesis remains understudied due to the biosafety and logistical challenges of such research. By adapting CyTOF and scRNA-seq approaches for use in BSL-4 containment, we comprehensively surveyed the molecular correlates of disease progression and viral replication in circulating immune cells in a NHP model of EVD. This study, which is the first high-parameter, single-cell investigation under BSL-4 containment, shed new light on changes in peripheral cell-type and -state abundance throughout lethal EVD, characterized the EBOV-infected populations of circulating immune cells, and identified genes regulated by the cytokine milieu or by direct EBOV infection.

We characterized transcriptional- and protein-level changes in monocytes during EVD in NHPs, some of which reflect disruption of their physiological antiviral function. In agreement with the well-established importance of monocytes in EVD (Menicucci et al., 2017), we observed that they had over twice as many differentially expressed genes as other cell types, including genes involved in IFN response, cytokine production, myeloid differentiation, and antigen presentation. Monocytes became activated by IFN during EVD, which normally upregulates MHC class II genes (Steimle et al., 1994). Surprisingly, almost all MHC class II and related antigen-presentation genes were strikingly downregulated. Moreover, MHC class II expression decreased in both infected and uninfected monocytes, suggesting that the decrease was caused by the cytokine milieu. Reduced monocytes antigen presentation might explain why a failed or delayed adaptive immune response is a hallmark of fatal EVD in humans (Baize et al., 1999; Lüdtke et al., 2016).

As EVD progressed, conventional CD14^+^ and CD16^+^ monocyte subsets largely disappeared and were replaced by two populations: CD14^+^ CD16^+^ (DP) monocytes, which increase in other infections (Michlmayr et al., 2018; Zanini et al., 2018a), and an unexpected CD14^–^ CD16^–^ (DN) population, that, to our knowledge, has not been previously described in viral infection. The DN monocytes were highly proliferative, with transcriptomes more similar to bone marrow monocyte precursors than circulating monocytes. This suggests that they are the product of emergency myelopoiesis, in which cytokines stimulate the bone marrow to release immature myeloid-lineage cells (Chiba et al., 2018; Hérault et al., 2017; Al Sayed et al., 2019). DNs had high expression of neutrophil granule genes such as *MPO* and *AZU1*, suggesting that they represent myeloid progenitors prior to the branching of neutrophil and monocyte lineages. We also identified the emergence of an analogous DN population in human acute EVD cases. This finding highlights the power of high-parameter methods such as scRNA-seq and CyTOF: despite little-to-no detection of CD14 or CD16, there were enough other RNA and protein markers to reliably identify these DNs as monocyte precursors.

Our data refine the picture of EBOV’s tropism in NHPs, demonstrating that the predominant infected population in circulation are DP monocytes expressing markers of macrophage differentiation. Myeloid cells are known to be major targets of EBOV (Geisbert et al., 2003b, 2003c), including DCs *ex vivo* and in lymph nodes *in vivo* (Geisbert et al., 2003c). While we too observe infected DCs *ex vivo*, *in vivo* we only detected infected monocytes among circulating immune cells. This might reflect biological phenomena required for DC infection, such as cell density, cell-to-cell contact, or viral dose. Viral tropism heavily depends on viral entry, during which the cellular receptor NPC1 and other entry factors are required. While we did not observe differences in *NPC1* mRNA expression between infected and uninfected monocytes, expression of known EBOV entry factors like cathepsin B and L (Chandran et al., 2005; Martinez et al., 2013; Schornberg et al., 2006) were associated with infection. Cultured monocytes are only susceptible to EBOV infection upon differentiation (Martinez et al., 2013), and our data further show that infectivity *in vivo* strongly correlates with physiological variability of monocyte differentiation state. Furthermore, our data show that the relative abundance of DP monocytes, the preferred circulating cell targets of EBOV, increases over the course of infection, perhaps driven by the cytokine milieu of EVD.

Among infected monocytes, we observed substantial heterogeneity in intracellular viral load (up to 57.5% of transcripts), which we exploited as a proxy for staging EBOV’s progression through its life cycle. This enabled us to better understand the over-representation of *GP* mRNA that had been observed in bulk RNA-seq datasets (Menicucci et al., 2017). In our data, cells with low viral load (reflecting early infection) had EBOV transcript abundances that mirrored the genome organization as expected (Brauburger et al., 2014, 2015)—i.e., expression was highest for *NP* and lowest for *L*. But cells with high viral load had higher abundance of *GP* than *NP* mRNA, suggesting that alternate transcription or post-transcriptional regulation, such as increased mRNA stability, may account for accumulation of *GP* late in the viral life cycle. Translation of GP, a structural protein, at the end of the viral life cycle coincides with virion assembly and thus may benefit EBOV fitness, as has been observed for other viruses (Honess and Roizman, 1974; Irigoyen et al., 2016; King et al., 2018; Shin et al., 2015).

Many host ISGs negatively correlated with intracellular viral load, and our data suggest that viral infection downregulates ISG expression *in vivo*, rather than preferentially infecting cells with low ISG levels. First, there are multiple established mechanisms by which EBOV downregulates ISGs, such as by preventing STAT1 from translocating to the nucleus (Reid et al., 2006). Second, monocytes mount a strong ISG response by DPI 3, yet the percentage of infected cells rises gradually from DPI 4 onward, implying that EBOV is able to replicate despite the inhibitory activities of ISGs. Third, we do not observe any cells with low (0.01%–0.1%) viral load and low ISG levels. Since a newly infected cell begins with low viral load, this observation implies that EBOV is infecting monocytes that are mounting a full IFN response. Because EBOV potently suppresses IFN production in infected cells, it remains unclear how the IFN response is triggered. Possibilities include incomplete IFN antagonism in infected cells, defective particles, or genomes that fail to suppress IFN (Calain et al., 1999, 2016; Russell et al., 2019), pathogen- and danger-associated molecular patterns released from dying infected cells (Cárdenas et al., 2006; Reynard et al., 2019), and other classes of cytokines produced from cells that do not support EBOV replication (Harcourt et al., 1999; Rhein et al., 2015). Better understanding this antiviral response could be the key to novel therapeutics or vaccines (Woolsey et al., 2019).

Several putative pro-viral host genes were *positively* correlated with intracellular viral load, suggesting that they are directly responding to, or are regulated by, the presence of virus in infected cells (Figures 7A, 7B, and 7D). *DYNLL1* was the gene most associated with viral load. Previous work identified an interaction between DYNLL1 and EBOV VP35 (Kubota et al., 2009) that increased EBOV RNA synthesis in a minigenome assay (Luthra et al., 2015). DYNLL1 increases replication of rabies virus (Tan et al., 2007) an NNS virus similar to EBOV. Here, we showed that *DYNLL1* expression is upregulated within infected cells *in vivo*, suggesting that EBOV manipulates cellular pathways to encourage a pro-viral cellular environment. Nuclear DYNLL1 typically represses its own transcription factor ATMIN (Jurado et al., 2012); we hypothesize that EBOV VP35 sequesters DYNLL1 protein in the cytoplasm, relieving repression of ATMIN, thus upregulating *DYNLL1* mRNA. Additional genes that were upregulated with intracellular viral load were associated with translation and cell stress (e.g., *HSPA5*, *IARS)*, reflecting the strain placed on cells by viral replication. By computationally staging cells within the viral life cycle, we nominated several putative pro-viral genes for further study, highlighting the utility of single-cell profiling to study host-virus interactions.

Our data provide insights into circulating immune cells in a lethal model of EVD in rhesus monkeys; however, there are several key aspects of pathogenesis that are not reflected in this study. Much of the clinical syndrome of EVD is due to viral replication and pathology in tissues, including the liver, vasculature, lung, kidney, lymph nodes, and bone marrow (Martines et al., 2015). Studies of these compartments would be complementary to ours as immune cells pass through the circulation and extravasate into tissues to fight infection. An additional limitation of our study is that human EVD is not always lethal and has slower clinical kinetics than the uniformly lethal model used here. Studying EVD in survivors, either in human clinical cases or in non-uniformly lethal animal models, could substantially broaden our understanding of pathogenesis and successful adaptive immunity.

The accumulation of additional host-pathogen single-cell datasets promises to greatly enhance our understanding of infection by allowing us to determine which features of pathogenesis are shared between, or specific to, individual pathogens. For example, our scRNA-seq and CyTOF data identified several molecular commonalities between EVD and immunosuppressive septic shock (Bray and Mahanty, 2003), which is also characterized by loss of MHC class II expression in monocytes (Monneret and Venet, 2014; Reyes et al., 2020), increased DP monocytes (Fingerle et al., 1993; Nockher and Scherberich, 1998), and emergency myelopoiesis (Bomans et al., 2018; Cuenca et al., 2015; Reyes et al., 2020). Soluble mediators, including cytokines and glucocorticoids, could be key drivers of both EVD and sepsis pathophysiology. Tumor necrosis factor (TNF)-α signaling has been extensively implicated as a driver of systemic loss of vascular resistance and shock during EVD. Glucocorticoids have been less well studied in EVD but decrease MHC class II during sepsis (Hawrylowicz et al., 1994; Le Tulzo et al., 2004) and reduce *CD14* expression (Nockher and Scherberich, 1997) while increasing the abundance of DP monocytes (Liu et al., 2015). Indeed, the connection between EVD and sepsis may be direct: studies have found bacterial invasion during EVD in NHPs (Reisler et al., 2018) and in humans (Carroll et al., 2017), with immune signatures that resemble sepsis (Eisfeld et al., 2017).

In summary, this work expands our understanding of EVD and provides a general paradigm for exploring molecular features of host-pathogen interactions, such as tropism and dysregulation of cell circuitry in infected cells, which can be applied to other emerging pathogens.

## STAR★Methods

### Key Resources Table

**Table:** 

REAGENT or RESOURCE	SOURCE	IDENTIFIER
**Antibodies**		

See Data S1	(Bjornson-Hooper et al., 2019a, 2019b)	N/A

**Bacterial and Virus Strains**		

Ebola virus/H. sapiens-tc/COD/1995/Kikwit-9510621 (EBOV/Kikwit; GenBank accession MG572235.1; *Filoviridae: Zaire ebolavirus*)	BEI Resources	Cat#NR-50306

**Chemicals, Peptides, and Recombinant Proteins**		

Xpert Ebola Lysis Reagent	Cepheid	Cat#GXEBOLA-CE-50
EnGen SauCas9, 20 μM	(Yourik et al., 2019); NEB	Cat#M0654T
16% paraformaldehyde	Electron Microscopy Sciences	Cat#15710

**Deposited Data**		

EBOV NHP infection single-cell RNA-Seq	This paper	GEO: GSE158390
EBOV NHP infection CyTOF	This paper	FlowRepository: FR-FCM-Z2LX
Human cell atlas bone marrow scRNA-Seq	(Hay et al., 2018)	https://data.humancellatlas.org/
Human healthy PBMC scRNA-Seq	10X	https://support.10xgenomics.com/single-cell-gene-expression/datasets “Aggregate of 8 Chromium Connect channels and 8 manual channels,” “5k Peripheral blood mononuclear cells (PBMCs) from a healthy donor (v3 chemistry),” “5k Peripheral blood mononuclear cells (PBMCs) from a healthy donor (Next GEM),” “5k Peripheral blood mononuclear cells (PBMCs) from a healthy donor with cell surface proteins (v3 chemistry),” “5k Peripheral blood mononuclear cells (PBMCs) from a healthy donor with cell surface proteins (Next GEM),” “10k PBMCs from a Healthy Donor - Gene Expression and Cell Surface Protein,” “10k PBMCs from a Healthy Donor (v3 chemistry)”
EBOV human infection CyTOF	(McElroy et al., 2020)	Author correspondence

**Oligonucleotides**		

Primer: dN-SMRT: AAGCAGTGGTATCAACGCAG AGTGANNNGGNNNB	(Hughes et al., 2020); IDT	N/A
gRNA sequence: DASH_SeqB: GGGNNNNAAGC AGUGGUAUCAACGGUUAUAGUACUCUGGAAA CAGAAUCUACUAAAACAAGGCAAAAUGCCGUG UUUAUCUCGUCAACUUGUUGGCGAGAU	This paper	N/A

**Software and Algorithms**		

DropSeqPipe	(Macosko et al., 2015)	https://github.com/Hoohm/dropSeqPipe
Scanpy	(Wolf et al., 2018)	https://github.com/theislab/scanpy
MAST	(Finak et al., 2015)	https://github.com/RGLab/MAST
HARMONY	(Korsunsky et al., 2019)	https://pypi.org/project/harmony-pytorch/, https://github.com/immunogenomics/harmony
MAGIC	(van Dijk et al., 2018)	https://github.com/KrishnaswamyLab/MAGIC
cNMF	(Kotliar et al., 2019)	https://github.com/dylkot/cNMF
Scrublet	(Wolock et al., 2019)	https://github.com/AllonKleinLab/scrublet

### Resource Availability

#### Lead Contact

Further information and requests for resources and reagents should be directed to Aaron Lin (alin@broadinstitute.org).

#### Materials Availability

This study did not generate new unique reagents.

#### Data and Code Availability

The analysis scripts used in this study are available at https://github.com/dylkot/SC-Ebola.

The accession number for single-cell RNA-Seq datasets reported in this paper is GEO: GSE158390, with raw sequence data available on SRA.

The accession number for CyTOF .fcs files reported in this paper is FlowRepository: FR-FCM-Z2LX.

### Experimental Model and Subject Details

This study included a subset (21 of 27) outbred rhesus monkeys (*Macaca mulatta*) of Chinese origin described recently (Bennett et al., 2020; Greenberg et al., 2020). These 27 nonhuman primates (NHPs) were randomized into cohorts (Figure S1A, (Bennett et al., 2020)), balancing age, weight, and sex across 7 groups. All work was approved and performed in accordance with the Guide for the Care and Use of Laboratory Animals of the National Institute of Health, the Office of Animal Welfare, and the US Department of Agriculture (Bennett et al., 2020).

### Method Details

#### Serial sampling study

This study utilized the Ebola virus/*H. sapiens*-tc/COD/1995/Kikwit-9510621 (EBOV/Kikwit; GenBank accession MG572235.1; *Filoviridae: Zaire ebolavirus*) isolate for the *in vivo* and *ex vivo* challenges, obtained from the Biodefense and Emerging Infections Research Resources Repository (BEI Resources, Manassas, VA, USA). It is the standard challenge stock defined by the filovirus animal non-clinical group (FANG) for testing product efficacy for FDA approval and is well characterized.

For all 21 outbred rhesus monkeys, two baseline blood samples were collected between 0–14 and 14–30 days prior to infection (Figure S1A). 18 NHPs were exposed to the EBOV/Kikwit isolate diluted to a target concentration of 1,000 plaque forming units (PFU) in a volume of 1 mL/dose. All NHPs were inoculated within a 5 month period. This same cohort has already been described recently (Bennett et al., 2020; Greenberg et al., 2020).

#### Clinical observations and scoring

Beginning on day post-infection (DPI) 0, NHPs were observed 1–3 times daily and given a clinical score based on five criteria: overall clinical appearance and signs of hemorrhage; respiratory rate, mucous membrane color, and dyspnea; recumbency; non-responsiveness; and core temperature (Bennett et al., 2020). Each criterium was assigned a score between 1 and 10, and all scores were added together. Once an NHP reached a combined score of > 10, the animal was humanely euthanized.

#### Whole blood collection

Blood was drawn from anesthetized animals into BD vacutainer plastic serum separator tubes (SST) for serum viral load quantification, or in BD vacutainer plastic blood collection tubes with K_3_EDTA for hematology and peripheral blood mononuclear cell (PBMC) purification (Becton Dickinson, Franklin Lakes, NJ, USA) (Bennett et al., 2020). SST tubes were centrifuged at room temperature for 10 minutes (min) at 1800 x *g* to isolate serum. K_3_EDTA tubes were mixed by gentle inversion prior to hematology and PBMC purification.

#### Hematology and complete blood counts (CBC)

250 μL of each whole blood sample was analyzed on a Sysmex 2000i XT (Sysmex Corporation, Kobe, Hyogo Prefecture, Japan) (Bennett et al., 2020). Parameters analyzed by this instrument were: counts of basophils, eosinophils, lymphocytes, monocytes, neutrophils, white blood cell count; percentages of each cell type; and mean platelet volume.

To estimate the abundance of lymphocyte cell types, we multiplied the CBC lymphocyte count by the proportion of lymphocytes of each cell type (CD8 T cells, CD4 T cells, NK cells, and B cells) which was calculated from the unsupervised clustering of the CyTOF data (see ‘CyTOF’ below).

#### EBOV serum viral load by reverse transcription quantitative PCR

70 μL of sample inactivated by TRIzol LS was added to 280 μL of Buffer AVL (QIAGEN, Hilden, Germany) with carrier RNA (Bennett et al., 2020). Samples were then extracted using the QIAamp Viral RNA Mini Kit (QIAGEN) in accordance with the manufacturer’s instructions, eluted in 70 μL of Buffer AVE, aliquoted, and frozen. Viral load was determined using the BEI Resources Critical Reagents Program experimental EZ1 reverse transcription qPCR kit assay in accordance with the manufacturer’s instructions.

#### PBMC purification

We centrifuged whole blood in K_3_EDTA tubes at 1800 x *g* for 10 min at room temperature, removed EDTA plasma, and added phosphate buffered saline (PBS, Thermo Fisher Scientific, Waltham, MA, USA) to the pelleted cells to double the original whole blood volume. We gently poured the PBS-blood cell mixture into an Accuspin tube containing Histopaque (Sigma-Aldrich, St. Louis, MO, USA) and centrifuged at 1000 x *g* for 10 min at room temperature with the brake set to 1. Following centrifugation, we removed the top, clear supernatant layer to within 0.5 cm of the cloudy white layer containing PBMCs.

We transferred the cloudy PBMC layer to a clean 15 mL conical tube and increased the volume to 10 mL using PBS supplemented with 2% heat-inactivated fetal bovine serum (PBS/2%HI-FBS) and mixed by inversion. We then centrifuged at 300 x *g* for 10 min at 4°C with the brake set to 1. Following centrifugation, we removed the supernatant, resuspended the cell pellet with PBS/2%HI-FBS to a final volume of 10 mL, and mixed using gentle raking to wash the cells. We repeated the wash step 2 more times with the centrifuge set to 200 x *g* for 10 min at 4°C with the brake set to 1. We then resuspended the cell pellet in 9.5 mL PBS/2%HI-FBS for counting using the Countess Cell Counting system (Thermo Fisher Scientific). We aliquoted 0.5 mL for Seq-Well, and used the remaining 9 mL volume for CyTOF.

#### Seq-Well

We performed Seq-Well as described previously (Gierahn et al., 2017), with the S^3^ protocol (Hughes et al., 2020) and some controls and modifications to adhere to the BSL-4 environment.

As an experimental negative control to test our statistical model, we spiked Madin-Darby canine kidney (MDCK) cells, constituting ∼5% of the total sample, into a subset of PBMC samples (Table S1) from EVD NHPs immediately before scRNA-Seq. As MDCKs were not exposed to EBOV, viral reads in these transcriptomes should be due to ambient RNA contamination (Russell et al., 2019).

After loading and sealing beads and cells in Seq-Well arrays, we placed them in a −80°C freezer until further processing – this step was required due to time constraints in the BSL-4. Later, we removed sealed Seq-Well arrays from the −80°C freezer, placed them in 4-well dishes, and allowed them to equilibrate to room temperature for at least 30 min. We then covered arrays in 5 mL Seq-Well Lysis Buffer per protocol.

We performed RNA hybridization and RT as specified in the protocol (Hughes et al., 2020). After RT, we collected beads by centrifugation at 1000 x *g* for 1 min at room temperature. We resuspended beads with GeneXpert Lysis Buffer (Cepheid, Sunnyvale, CA, USA) for inactivation, which was required prior to removal from the BSL-4 laboratory according to standard operating procedures. After removal, we washed beads thrice with TE buffer containing 0.01% Tween 20 and shipped at 4°C for further library construction and sequencing, which was performed with the S^3^ protocol (Hughes et al., 2020). We sequenced all libraries on either NextSeq 550 High Output or NovaSeq 6000 S2 flowcells (Illumina, San Diego, CA, USA), with 20 cycles for Read 1 (cell barcode and unique molecular index [UMI]) and 88 cycles for Read 2 (cDNA of interest). In some cases, we merged fastq files from multiple sequencing runs for increased coverage.

#### Depletion of abundant sequences by hybridization (DASH) of select Seq-Well libraries

We observed long concatemers of the common scRNA-Seq adaptor sequence (SeqB, 5′-AAGCAGTGGTATCAACGCAGAGTAC-3′) at high frequency in some Seq-Well libraries, likely owing to low RNA input and the challenging environment of processing samples in the BSL-4 suite. These concatemers disrupted Illumina sequencing runs because the Read 1 sequencing primer annealed to multiple SeqB sequences on a single template, allowing multiple sequencing-by-synthesis reactions simultaneously. We therefore devised a strategy to remove SeqB concatemers using depletion of abundant sequences by hybridization (DASH) (Gu et al., 2016), a CRISPR-based method to degrade target DNA sequences prior to sequencing. SeqB lacked a ‘NGG’ protospacer adjacent motif (PAM) for the common *S. pyogenes* Cas9 (SpyCas9) for which DASH was originally described; therefore, we modified DASH to use *S. aureus* Cas9 (SauCas9) (Ran et al., 2015). Moreover, in contrast to SpyCas9, SauCas9 is a multi-turnover enzyme (Yourik et al., 2019), suggesting that SauCas9 would have higher cleavage efficiency, which was important since SeqB was present in multiple copies within a concatemer.

First, we designed a guide RNA (gRNA) to target SeqB. Based on the position of the SauCas9 PAM and the length of SeqB, only 17 nucleotides of the gRNA protospacer could anneal to SeqB. Because gRNA length is critical to SauCas9 cleavage efficiency (Friedland et al., 2015; Ran et al., 2015), we prepended 4 random bases as a 5′ overhang (Key Resources Table). We *in vitro* transcribed this gRNA using the MEGAshortscript T7 Transcription Kit (Thermo Fisher Scientific), purified it using the RNA Clean & Concentrate Kit (Zymo Research, Irvine, CA, USA), and verified the correct RNA length on a 15% TBE-urea gel (Bio-Rad Laboratories, Hercules, CA, USA).

We performed SauCas9 DASH according to reaction conditions laid out for *in vitro* SauCas9 cleavage assays (Yourik et al., 2019). We incubated 10 pmol gRNA with 5 pmol SauCas9 (New England Biolabs [NEB], Ipswich, MA, USA) at 25°C for 10 min, and then added up to 5 fmol DNA (2000:1000:1 RNA:protein:DNA ratio) and NEBuffer 3.1 (NEB) to 1X. We incubated this reaction at 37°C for 2 hours (h), and quenched by adding EDTA to 50 μM, SDS to 1%, and 4 total U of Proteinase K (NEB) at room temp for 10 min. We removed degraded concatemers with two consecutive 0.8X SPRI purifications using Ampure XP beads (Beckman Coulter, Brea, CA, USA), eluted, and performed 6–9 cycles of PCR with the NEBNext Ultra II Q5 Master Mix (NEB) using Illumina P7 and the Seq-Well P5-TSO hybrid primer (Gierahn et al., 2017).

#### CyTOF

We added 1 mL of 16% paraformaldehyde (PFA, Electron Microscopy Sciences, Hatfield, PA, USA) to 9 mL PBMCs to fix the cells. We incubated samples at room temperature for 10 min followed by a final centrifugation at 600 x *g* for 5 min at 4°C with the brake set to 9. We removed the supernatant, added 1 mL of PBS/5%HI-FBS for every 3 × 10^6^ cells (e.g., 2 mL for a sample containing 6 × 10^6^ cells), aliquoted into 1 mL aliquots in cryovials, and stored in a −80°C freezer.

We equilibrated fixed PBMCs to come to room temperature before transferring approximately 2 × 10^6^ cells per sample into 1.2 mL cluster tubes in a 96-tube rack. We barcoded samples and multiplexed them into 6 batches of 16 samples using a previously described 16-plex palladium-based mass-tag cell barcoding scheme (Zunder et al., 2015). We aspirated pelleted barcoded cells to a volume of 50 μL and incubated them with 15 μL of Human TruStain FcX (Biolegend, San Diego, CA) for 10 minutes. We stained cells for 30 min with 175 μL of a reconstituted lyophilized cocktail of metal-tagged cell-surface antibodies described previously (Bjornson-Hooper et al., 2019a, 2019b) supplemented with two additional antibody channels (Data S1). We washed and permeabilized surface-stained cells with methanol before aspirating down to 50 μL and staining for 60 min with 190 μL of reconstituted lyophilized intracellular staining antibody cocktail (Data S1). We washed and resuspended fully-stained cells in a volume of 750 μL.

Following staining, we inactivated samples by adding 250 μL of 16% PFA to 750 μL of each sample, for a final concentration of 4% PFA, and incubated at 4°C overnight. The next day, we centrifuged samples 600 x *g* for 5 min at 4°C and aspirated down to 100 μL. We resuspended samples in 1 mL 4% PFA in PBS and transferred to a clean 2 mL cryovial. We then removed samples from the BSL-4 using a dunk tank and froze at −80°C within 30 min of PFA addition.

Following inactivation, we thawed and processed inactivated samples within a BSL-2 lab for iridium intercalation, then mixed with 1xEQ beads (Fluidigm, South San Francisco, CA, USA) and run on a CyTOF Helios (Fluidigm) instrument using a Super-Sampler introduction system (Victorian Airship & Scientific Apparatus LLC, Alamo, CA, USA).

FCS data files were normalized across all runs using the data normalization software (Finck et al., 2013) and debarcoded using the single-cell debarcoder tool (Zunder et al., 2015) as previously described. Data were uploaded and analyzed using CellEngine software (https://cellengine.com, Primity Bio, Fremont, CA, US), and a gating strategy was applied to identify cell populations using canonical markers. Frequencies for each population were determined as a function of total CD66-CD45+ cell events and reported marker intensities are expressed as medians. Also see ‘CyTOF data preprocessing, clustering, and dimensionality reduction’ section below for details of unsupervised analysis of CyTOF data.

#### *Ex vivo* inoculation of PBMCs

PBMCs were isolated from healthy NHPs as previously described. We diluted cells to 3.3 × 10^6^ cells/mL RPMI/10%HI-FBS and transferred 900 μL each to 2 mL external thread cryogenic vials (Corning, Corning, NY) for each experimental condition. We inoculated cells with 100 μL live virus (EBOV/Kikwit, the same stock used for *in vivo* NHP inoculation) for a final MOI of 0.1 PFU/cell, an equivalent dose of irradiated virus (EBOV/Kikwit treated with 5 mRads gamma irradiation), or media only, and incubated for 4 or 24 hours with slow rocking prior to Seq-Well processing.

#### Single-cell RNA-Seq raw data processing

Raw sequencing files were demultiplexed and converted to fastq using bcl2fastq version 2.20. Reads were then trimmed, aligned to a reference transcriptome, and parsed into a digital gene expression matrix using the previously published Dropseq-tools pipeline (Macosko et al., 2015) version 2.0. We used a Snakemake wrapper around Dropseq-tools that is available in the open source Github repository https://github.com/Hoohm/dropSeqPipe. In brief, we trimmed adaptor sequences using Cutadapt (Martin, 2011) version 1.16, performed spliced alignment of trimmed reads using STAR aligner (Dobin et al., 2013) version 2.6.1b, identified core barcodes using the whitelist function in umi_tools (Smith et al., 2017) version 0.5.5, and used Dropseq-tools to correct barcodes and extract digital count matrices. A frozen version of the pipeline used to process the Seq-Well data is available at the Github repository https://github.com/dylkot/dropSeqPipe-dak.

Sequencing reads were aligned to a hybrid genome/genebuild of *Macaca mulatta* (genome assembly Mmul_8.01, Ensembl gene build 92) and EBOV/Kikwit (GenBank accession KU182905.1).

#### Single-cell RNA-Seq data preprocessing, clustering, dimensionality reduction, and smoothing

The scRNA-Seq data was preprocessed, clustered, and visualized using Scanpy (Wolf et al., 2018). We removed cells with < 300 genes detected, > 10% of their UMIs derived from mitochondrial genes, or > 95% of UMIs mapped to non-genic regions. We excluded ribosomal genes, genes correlated with the percentage of UMIs assigned to mitochondrial genes (Pearson R > 0.1), and *HBB* as these were largely driven by the technical covariate of whether cells had loaded into Seq-Well arrays fresh or had undergone a freeze-thaw cycle with cryoprotectant. We also excluded EBOV genes and cell-cycle genes (defined by correlation with *TOP2A,* Pearson R > 0.1) prior to clustering so that these signals would not influence identification of cell types.

We performed multiple iterations of clustering to detect and exclude doublets and to identify distinct cell populations at multiple levels of granularity. In each iteration, we filtered genes detected in < 10 of the cells being clustered. We then transformed raw UMI counts by normalizing the sum of counts of each cell to 10,000 (TP10K), adding 1 to each expression value, and taking the natural logarithm. In each clustering iteration, we identified and subsetted the data to highly variable genes using the highly_variable_genes function in Scanpy (Satija et al., 2015) with the default parameters. We *Z*-normalized each gene and set transformed values exceeding 10 to 10. *Z*-normalized data was used as input to principal component analysis. We determined the number of principal components to use for downstream analysis by identifying an elbow on the Skree plot of the eigenvalues associated with each principal component. For the *in vivo* Seq-Well data, we used the Harmony algorithm (Korsunsky et al., 2019) to remove variation due to whether a PBMC sample had been processed fresh, or following a freeze-thaw. No Harmony adjustment was used for the *ex vivo* EBOV dataset. The Harmony-adjusted or raw principal components were then used to construct a nearest neighbor graph with the number of neighbors set to the maximum of 30 or 0.001 x the number of cells. Lastly, we clustered cells using the Leiden community detection algorithm (Traag et al., 2019).

We annotated broad PBMC clusters in the *in vivo* and *ex vivo* EBOV datasets based on the following marker genes: CD8+ T cells (*CD3D*, *GZMB*, *GNLY*), CD4+ T cells (*CD3D*, *IL7R*), B cells (*MS4A1*, *IGHM*), Monocytes (*CFD*, *LYZ*), cDCs (*FLT3*, *IRF8*), pDCs (*IRF8*, *GZMB*), Neutrophils (*CD177*, *LCN2*), Platelets (*PF4*, *CAVIN2*), Plasmablasts (*MZB1*, *IGHM*, *IGHA*), and spike-in control MDCK cells (*COL5A2*, *SLC20A1*).

For the *in vivo* EBOV dataset, the first clustering iteration was used to identify and filter a cluster of multiplets (expressing high levels of B cell, T cell, Neutrophil, and Monocyte genes) and MDCK control cells. A second clustering iteration was run on the filtered data to identify broad cell type clusters of T/NK-cells, B cells, and myeloid cells (Monocytes, cDCs, pDCs, and neutrophils). Sub-clustering was performed on each broad cell type in two iterations, the first to identify remaining doublets to exclude, and the second to cluster cells into final cell-types and sub-types based on annotation of marker genes (Figure S2).

An analogous sequence of clustering iterations was used for the *ex vivo* EBOV dataset. However, as there were no MDCK cells spiked in, we proceeded straight to sub-clustering of the T/NK, B, Monocyte/DC, and multiplet populations from the initial clustering iteration.

Doublets and multiplets identified during any clustering iteration were excluded. Then visualization in 2 dimensions was accomplished by computing the nearest neighbor graph with 0.001 x the number of cells as nearest neighbors, followed by Uniform Manifold Approximation and Projection (Becht et al., 2018).

Gene expression values were smoothed to facilitate direct visualization of *CD14*, *FCGR3* (which codes for CD16), and *MKI67* (which codes for Ki67) by running MAGIC (van Dijk et al., 2018) on log TP10K expression values with the ‘cosine’ distance metric and 3 diffusion steps.

#### Differential expression testing

We performed all differential expression tests using MAST (Finak et al., 2015) on log(TP10K + 1) normalized data. For all differential expression tests, we included (1) the percentage of mitochondrial reads and (2) the number of genes detected in a cell as covariates. For tests in the *in vivo* dataset, we additionally included a binary indicator covariate of whether the cell was derived from a sample that had been processed fresh, or had undergone freeze-thaw. For tests in the *ex vivo* dataset, we additionally included a binary indicator covariate of which NHP donor the sample was derived from.

For viral load comparisons, we used the log10 viral load as a continuous exogenous variable and only considered cells with ≥ 1 viral read. For all other comparisons, we used a binary exogenous variable indicating the reference and the query group. “Viral load” and “bystander vs. EBOV infected cells” in the *in vivo* dataset were conducted only considering cells from DPI 5–8. “Viral load” comparisons in the *ex vivo* dataset only considered cells from the 24 HPI time point.

Differential expression *p*-values were corrected for multiple hypothesis testing using the method of Benjamini and Hochberg (Benjamini and Hochberg, 1995).

#### Identifying differential gene expression modules

We clustered the log fold-change profiles of 1,437 differentially expressed genes (rows of Figure 3A) considering B, CD4+ T, CD8+ T, NK, and monocyte populations at the three EVD stages. Prior to clustering, insignificant values (p > 0.2) were first set to 0 and genes were normalized to unit L2 norm. We performed *k*-means clustering with K = 11 and the default parameters in Scikit-learn (Pedregosa et al., 2011) version 0.22.2.post1. Varying K above and below 11 led to highly consistent results with splitting or merging individual clusters at the margin, and we picked K = 11 as the lowest value that yielded the “B down” module.

#### Detection of EBOV infected cells

Our infection detection model assumes that EBOV reads assigned to a cell are either due to true viral RNAs inside that cell, or due to ‘ambient’ extracellular EBOV RNA (Fleming et al., 2019; Young and Behjati, 2018) in the sample that, by chance, are captured in a well of the Seq-Well array along with the cell. Our null model for how many EBOV reads would be expected in a cell by chance therefore depends on two main parameters which we estimate from the data: (1) what proportion of ambient RNAs in a sample are due to EBOV, and (2) what proportion of a cell’s expression profile is due to ambient RNA. Our method therefore precedes through the following steps which we describe below:

1 Estimate an ambient RNA profile for each Seq-Well array
2 Estimate the proportion of each cell’s transcripts that are due to ambient RNA
3 Determine if there are more EBOV transcripts in each cell than would be expected based on the proportion of EBOV in the ambient RNA and the cell’s estimated level of ambient RNA contamination.

#### 1. Estimate an ambient RNA profile for each Seq-Well array

We assume that cell barcodes with few transcripts detected correspond to wells in the Seq-Well array that lack a cell, and therefore, that any transcripts assigned to those cell barcodes derive from ambient RNA. We consider all cell barcodes with fewer than 50 transcripts detected to be empty wells and compute an ambient RNA profile of the proportion of transcripts from these barcodes assigned to each gene. This is analogous to the approach used in several ambient RNA correction approaches such as (Fleming et al., 2019).

We denote the number of genes in the dataset as *G* and define the ambient RNA profile for a given Seq-Well array, *a*, as a *G*-dimensional vector $P^{\left(a\right)}$. $P_{g}^{\left(a\right)}$denotes the estimated proportion of ambient RNAs that are assigned to gene *g* based on transcripts from all cell barcodes with fewer than 50 UMIs. Since $P_{g}^{\left(a\right)}$is a proportion, the following constraints hold:

$$0<P_{g}^{\left(a\right)}<1;\sum_{g=1}^{G}P_{g}^{\left(a\right)}=1$$

#### 2. Estimate the proportion of each cell’s transcripts that are due to ambient RNA

We adapt the previously published Consensus Non-negative Matrix Factorization (cNMF) method (Kotliar et al., 2019) to estimate the ambient RNA contamination level of each cell. In brief, cNMF learns a user-specified number of gene expression programs (GEPs), each a non-negative *G*-dimensional vector representing the average expression profile of an individual cell type or cellular activity (e.g., cell-cycle or interferon response) that are present in the data. In addition, it learns a “Usage” matrix reflecting the % contribution of each GEP in each cell (i.e., the % of each cell’s transcripts derived from each GEP). For the following notation, matrices are indicated in bold. Denoting the number of cells in the dataset as *C* and the user-specified number of GEPs as *K*, cNMF is given an input *C*x*G* matrix of transcript counts $X_{cg}$ and returns $G_{kg}$ a non-negative *K*x*G* matrix of GEPs reflecting the relative contribution of gene *g* in GEP *k*, as well as a non-negative *C*x*K* matrix $U_{ck}$ reflecting the percentage of transcripts in cell *c* that are due to GEP *k*.

We adapt this approach to return an updated, *C*x(*K+1*) dimensional usage matrix that includes an additional column reflecting the usage of the ambient RNA profile. We run cNMF as published to obtain the $G_{kg}$ GEP matrix. We then append the ambient RNA profile for a given array as an additional row to $G_{kg}$ and normalize each row to sum to 1 like so:

$$\begin{array}{l} {\tilde{G}}_{i}=\frac{G_{i}}{{\left|G_{i}\right|}_{1}}\\ {\tilde{G}}^{\left(a\right)}=\left[\begin{array}{l} {\tilde{G}}_{1}\\ {\tilde{G}}_{2}\\ \vdots \\ {\tilde{G}}_{K}\\ {\tilde{P}}^{\left(a\right)} \end{array}\right] \end{array}$$

where ${∥}_{1}$ denotes the L1 norm, a tilde is used to denote an L1 normalized vector, and $G_{i}$ denotes the ith row of $G_{kg}$. We then run a final iteration of NMF with the GEP matrix fixed to ${\tilde{G}}^{\left(a\right)}$. This jointly estimates a usage of the ambient RNA profile and the other GEPs using non-negative least-squares. For a given cell *c* from an array *a*, this amounts to solving the following non-negative least-squares optimization:

$$\underset{U_{c1}^{\left(a\right)}...U_{c\left(K+1\right)}^{\left(a\right)}\geq 0}{min}\;{\left\|X_{c}-\sum_{k=1}^{K+1}U_{ck}^{\left(a\right)}\;{\tilde{G}}_{k}^{\left(a\right)}\right\|}_{2}$$

We combine these coefficients for all cells and programs into a single matrix and L1 normalize the usages to sum to 1:

$$\begin{array}{l} U^{\left(a\right)}=\left[\begin{array}{ccc} U_{11}^{\left(a\right)} & \cdots & U_{1\left(K+1\right)}^{\left(a\right)}\\ \vdots & \ddots & \vdots \\ U_{C\left(a\right)1}^{\left(a\right)} & \cdots & U_{C\left(a\right)\left(K+11\right)}^{\left(a\right)} \end{array}\right]\\ {\tilde{U}}^{\left(a\right)}=\left[\begin{array}{l} \frac{U_{1}^{\left(a\right)}}{{\left|U_{1}^{\left(a\right)}\right|}_{1}}\\ \vdots \\ \frac{U_{C\left(a\right)}^{\left(a\right)}}{{\left|U_{C\left(a\right)}^{\left(a\right)}\right|}_{1}} \end{array}\right] \end{array}$$

where C(a) denotes the number of cells derived from array *a*. The “K+1”th column of ${\tilde{U}}^{\left(a\right)}$ reflects the estimated proportion of transcripts due to ambient RNA in all of the cells from array *a*. We repeat this calculation for each array separately and denote the estimated contribution of ambient RNA for a cell *c* as $A_{c}$.

#### 3. Determine if there are more EBOV transcripts in each cell than would be expected by chance

For each cell, we determine a threshold, $T_{c}$, for the number of EBOV transcripts required to call that cell infected while keeping the false positive rate below *f*, a user specified threshold (*f =* 0.01 in all of our analyses). We first calculate $E^{\left(a\right)}$, the proportion of ambient RNA transcripts in array *a* that are due to EBOV as follows:

$$E^{\left(a\right)}=\sum_{g\in \left\{EBOV\;genes\right\}}P_{g}^{\left(a\right)}$$

Then, for a cell with $N_{c}$ transcripts total; a given proportion, $A_{c}$, of its reads derived from ambient RNA; $E^{\left(a\right)}$, the proportion of ambient RNA contaminating reads expected to map to EBOV, we compute $T_{c}$ using binomial statistics as follows:

$$T_{c}=F\left(f\;|\;p=E^{\left(a\right)}A_{c},\;N=N_{c}\right)$$

Where *F* is the inverse survival function of the Binomial distribution with event probability *p* and *N* trials. We identify cells with $>T_{c}$ reads as infected.

#### Estimation of the infection receiver operator characteristic

We estimate sensitivity to call an infected cell with either 1% or 0.1% of its reads due to EBOV across a range of 60 false positive rate thresholds (*f*). We first randomly sampled 2,000 cells from the live EBOV treatment samples in the *ex vivo data,* or the non-baseline samples from the *in vivo* data, to serve as an empirical distribution for $A_{c}$ and $N_{c}$. For each cell and specificity threshold *f*, we then calculate the probability of correctly calling a true positive cell as positive. We model the distribution of the number of EBOV reads in a cell as the convolution of 2 binomial distributions: $BN\left(p_{cell},\;N_{cell}\right)$is the binomially distributed number of true cell-derived reads mapping to EBOV and $BN\left(p_{ambient},\;N_{ambient}\right)$is the analogous distribution for ambient RNA-derived reads mapping to EBOV. $p_{cell}$ is 0.01 or 0.001 by assumption, and $p_{ambient}$ is estimated empirically for each cell as $E^{\left(a\right)}A_{c}$. We calculate the expected values for $N_{cell}$ and $N_{ambient}$ and round $N_{ambient}$ up to the nearest integer, as follows:

$$\begin{array}{l} N_{ambient,\;c}=Ceiling\left(N_{c}A_{c}\right)\\ N_{cell,\;c}=N_{c}-N_{ambient,\;c} \end{array}$$

We then directly calculate the convolution of the two binomial distributions for each cell:

$$P_{convolution}\left(X_{c}=x\right)=\sum_{j=0}^{x-i}\sum_{i=0}^{x}BN\left(i\;|\;p=p_{cell},\;N=N_{cell,\;c})BN(j\;|\;p=p_{ambient,\;c},\;N=N_{ambient,\;c}\right)$$

We then calculate the sensitivity for each cell as the probability that the convolution distribution is greater than the empirical threshold:

$$Sensitivity_{c}=P_{convolution}\left(X_{c}>T_{c}\right)$$

We plot the average sensitivity across the 2000 randomly sampled cells as a function of *f* for $p_{cell}$ equal to either 0.01 or 0.001.

#### Gene set enrichment testing

We downloaded gene sets from the Molecular Signatures Database (Liberzon et al., 2011) version 6.2 for gene set enrichment testing. We considered all Hallmark or C2 gene sets containing greater than 10 genes that were present in our expression data. We tested expression modules for enrichment using Fisher’s exact test and corrected for multiple hypothesis testing using the method of Benjamini and Hochberg (Benjamini and Hochberg, 1995).

To test continuous expression profiles for gene set enrichment (Figure S4B), we used the rank-sum test comparing genes in the gene set to all genes not in the set.

#### Scoring cells for interferon response and macrophage differentiation

We identified 58 genes in the “Global up” module that were also included in one or more of the following gene sets from the molecular signatures database: HECKER_IFNB1_TARGETS, BROWNE_INTERFERON_RESPONSIVE_GENES, MOSERLE_IFNA_RESPONSE, HALLMARK_INTERFERON_ALPHA_RESPONSE, HALLMARK_INTERFERON_GAMMA_RESPONSE (Table S3). We then scored cells for the average expression of these genes using the score_genes function in Scanpy (Satija et al., 2015) with 58 control genes, as this was the number of genes in the ISG set, and otherwise default parameters.

We computed a macrophage score based on the set of 618 genes annotated as significantly up or downregulated during *in vitro* monocyte-to-macrophage differentiation (Dong et al., 2013) (Table S4**)**. We computed each cell’s macrophage score as the dot-product of its expression profile for the 618 genes (in log TP10K) with the log fold-change reported for each gene in (Dong et al., 2013). This effectively weights genes by both the direction and magnitude of their change during *in vitro* macrophage differentiation.

#### Comparison of EVD monocyte subsets with human bone marrow and PBMC data

We obtained all of the human PBMC datasets produced using v3 or v3.1 chemistry from the 10X website (Key Resources Table), aggregated them together, and processed the resulting dataset using the same pipeline as the NHP Seq-Well data. Briefly, we first filtered out genes detected in fewer than 10 cells before converting to log TP10K and performed PCA as described above. Then, we used Harmony (Korsunsky et al., 2019) to integrate out variation due to the different samples of origin and used 30 nearest neighbors for Leiden community detection (Traag et al., 2019) and UMAP dimensionality reduction (Becht et al., 2018) (Figure S5E). We did not perform any sub-clustering on this dataset.

We obtained Human Cell Atlas bone marrow data from the Human Cell Atlas data portal (Hay et al., 2018) and processed it according to the same pipeline as the NHP data with a few modifications. We filtered doublets prior to clustering by running Scrublet (Wolock et al., 2019) separately within each of 8 donor batches with an expected doublet rate parameter of 6%. We identified and excluded cell-cycle associated genes, as those with a Pearson correlation > 0.3 with *TOP2A*. We integrated data from the different donor batches using Harmony and used 30 nearest neighbors for Leiden community detection and UMAP dimensionality reduction. We performed 3 rounds of sub-clustering: First we clustered all of the cells to identify monocyte and dendritic lineage cells (Figure S5F). Second, we clustered just hematopoietic stem cells (HSCs) and monocyte/dendritic progenitor cells to identify doublets (as those falling into a cluster characterized by T cell marker genes such as CD3D and CD3E). Finally, we re-clustered this set with the doublets excluded to identify monocyte lineage cells, plasmacytoid dendritic cells, and conventional dendritic cells (Figure S5G).

We confirmed our marker gene-based annotations of the myeloid cell populations by comparing these cells to the circulating human PBMC dataset. We identified the nearest neighbor of each bone marrow myeloid progenitor cell in the PBMC dataset based on Euclidean distance of TP10K-normalized cells, considering overdispersed genes identified in the PBMC dataset based on the V-score (baseline-corrected Fano factor) (Klein et al., 2015). We then visualized the nearest-PBMC assignment of the bone marrow myeloid cells on a UMAP embedding (Figure S5H).

Finally, we combined the monocytes and monocyte precursor cells from the human PBMC and bone marrow datasets into a single reference. We again normalized the data to log TP10K and computed UMAP embeddings following the same procedure as for the individual datasets (Figure S5I), using Harmony to remove variation due to donor sample. We then down-sampled this data so that there would be equivalent numbers of cells of each of the bone marrow and PBMC clusters (i.e., 982 cells per cluster as that was the number of cells in the smallest cluster). We identified the nearest neighbor of each NHP monocyte in the down-sampled reference dataset, as described above and computed the percentage of NHP monocytes assigned to CD14+ or CD16+ clusters from either human bone marrow or PBMC (Figure 5F).

#### Logistic regression prediction of EBOV infection *in vivo*

We used Statsmodels (Seabold and Perktold, 2010) version 0.11.1 to fit a logistic regression predicting EBOV infection status among all monocytes from late EVD, based on the following features: macrophage score, MAGIC smoothed values of *CD14*, and *CD16* (*FCGR3*), and an interaction term for the product of the MAGIC smoothed values for *CD14* and *CD16*.

#### CyTOF data preprocessing, clustering, and dimensionality reduction

Our clustering and dimensionality analysis of the NHP CyTOF data was analogous to the Seq-Well pipeline with a few adaptations. We down-sampled a total of 1.1 million cells, consisting of 300,000 baseline cells and 100,000 cells from each DPI, selecting a uniform number of cells from each sample at a given DPI. We used the Arcsinh transformation of CyTOF raw intensity values divided by 5, which is standard in the field. We set a ceiling of transformed intensity values for each gene at the 99.999th percentile to reduce the effect of very rare outliers. We mean-centered the data but did not variance normalize prior to PCA. We then performed multiple clustering iterations of the data using the Leiden algorithm with the number of nearest neighbors set to the maximum of 30 or 0.01% of the number of cells in the dataset.

We annotated broad PBMC clusters in the CyTOF datasets based on the following marker proteins: CD8+ T cells (CD3, CD8), CD4+ T cells (CD3, CD4), NK cells (CD16, CD161), B cells (CD19, IgM), Monocytes (CD11b, BDCA3, CD14, CD16, HLA-DR), cDCs (HLA-DR, CD11c, CD1c), pDCs (HLA-DR, CD4, CD123), Neutrophils (CD11b, CD66), Platelets (CD61, BDCA3), Plasmablasts (IgM high, but little or no CD19), Basophils (CD11b, CD123, low HLA-DR). There was also a cluster of cells characterized by high HLA-DR, Ki67, and CD38 which we annotate as “Unassigned APC.”

In the first clustering iteration, we identified and excluded clusters of doublets as those expressing markers of two or more broad cell-types. We also excluded a cluster characterized by high expression of CD3 but low expression of both CD4 and CD8 that we speculated was a technical artifact as it was nearly exclusive to a single CyTOF batch.

Next, to address batch effects between the different CyTOF runs, we grouped cells into the broad categories of Monocytes and DCs, CD4+ T, CD8+ T, NK, Neutrophils, B, Plasmablasts, and Platelets, and used COMBAT (Johnson et al., 2007) to adjust for batch effect separately within each broad category, using the default COMBAT parameters in the Scanpy implementation.

We then ran cNMF (Kotliar et al., 2019) to identify and regress out artifact signals in the data. We ran cNMF using all 42 markers as inputs and adapted the method to not perform any variance scaling prior to NMF; this is because CyTOF intensities of different markers are already expressed on a comparable scale, unlike genes in single-cell RNA-Seq data which can vary over different orders of magnitude. We also set a floor on the input data to be non-negative (because after COMBAT, a small percentage of the values were slightly below 0). We selected K = 7 as the cNMF dimensionality as the solution stability fell off dramatically at higher values. One gene expression program (GEP) was characterized by high levels of the platelet markers CD61 and BDCA3. It was mostly enriched in the platelet cluster but was also elevated in a subset of cells of all major cell types, which suggests that it reflects an artifact of platelets sticking to cells. A second GEP was characterized by high levels of all of the intracellular markers and it was also distributed throughout cells of multiple clusters. We interpreted this GEP as a cell permeabilization artifact reflecting the relative accessibility of a cell’s intracellular proteins to CyTOF antibody staining. We regressed these 2 GEPs out of the data by subtracting the matrix (outer) product of the Usage and GEP matrices. We denote the number of cells as *C*, the number of genes as *G* (42 in our data), and the number of programs selected as K (7 in our data). The *C*x*G* input data matrix is denoted as *X*, the *K*x*G* GEP matrix returned by cNMF as and the Cx*K* usage matrix as . Then the correction is as follows:

$$X^{corrected}=X-U^{platelet}x\;G^{platelet}-U^{perm}x\;G^{perm}$$

Where $U^{platelet}$ and $U^{perm}$are the *Cx*1 dimensional matrices representing the usage of the platelet and permeabilization GEPs and $G^{platelet}$ and $G^{perm}$are the 1x*G* dimensional matrices representing the spectra of the platelet and permeabilization GEPs, and multiplication is the outer product. We also filtered cells assigned to the platelet cluster in the initial clustering, since their predominant signal had been regressed out.

We then repeated clustering of the corrected data to identify broad cell types (Neutrophils, Monocytes, and DCs), followed by sub-clustering within each broad cluster to generate the sub-clusterings in Figure S2. The data was visualized using the UMAP algorithm as described for the scRNA-Seq data.

The human PBMC CyTOF data was processed with the same pipeline as the NHP data with a few modifications. The data were down-sampled to 280,000 cells total (20,000 per sample), batch correction was performed using Harmony, and 0.001 x the number of cells was used for K nearest neighbor graph construction. No cNMF or COMBAT adjustment steps were performed.

### Quantification and Statistical Analysis

Details of statistical testing, sample size, center, and dispersion can be found in the figure legends, the main text, and STAR Methods.

#### Additional Resources

This study did not generate new additional resources (website, forum, clinical trial).

## Supplemental Information

Table S1. Overview of Samples Analyzed by CyTOF and Seq-Well, Related to Figure 1 Description of each NHP PBMC sample profiled in the study for CyTOF (tab 1) and Seq-Well (tab 2). Includes the animal ID, day post infection, number of cells passing quality control filters, the processing batch, and other technical factors such as whether the sample was processed fresh or underwent freeze-thaw, was treated with DASH, or included an MDCK cell spike-in. Subsequent tabs for the final counts of each broad cell type cluster for CyTOF (tab 3) and Seq-Well (tab 4). Tab 5 contains the absolute count of cells in each sample in units of 1000 cells per microliter based on complete blood count (CBC).

Table S2. Differential Expression Profiles of Cell Types at Each EVD Stage Relative to Baseline, Related to Figure 3 Log fold-change, *p*-values, and *q*-values for MAST differential expression tests comparing gene expression levels of cells at each EVD stage relative to baseline, for each cell type.

Table S3. Gene-Set Enrichment Testing of Differential Expression Modules, Related to Figure 3 The “geneset_enrichment” tab includes the rank, gene set name, odds ratio (OR), and FDR-corrected q-value for the top 10 gene-sets associated with each differential expression module (ranked by q-value) based on Fisher’s exact tests. The “module_membership” tab includes the module assignment for all genes tested for differential expression including insignificant genes labeled as “Not significant.” The “ISG_set_forScoring” tab includes differentially expressed genes in the “global up” module that were annotated as interferon stimulated genes (ISGs) and subsequently used for computing the ISG score. The Unique_IFN_Alpha_Set species the genes included in the HALLMARK_INTERFERON_ALPHA_RESPONSE gene set but not the HALLMARK_INTERFERON_GAMMA_RESPONSE gene set, and Unique_IFN_Gamma_Set specifies the converse gene set.

Table S4. Differential Expression Results of Infected Versus Bystander Monocytes, *In Vivo*, at DPI 5–8, Related to Figure 5 Log fold-change, *p*-values, and *q*-values for differential expression tests comparing gene expression levels between infected and bystander monocytes in late EVD. Also includes annotations of whether each gene is associated with macrophage differentiation or interferon response. Subsequent tabs specify the published gene lists associated with *in vitro* macrophage differentiation that were used for gene set enrichment, as well as the contingency tables and hypothesis tests associated with each enrichment test.

Table S5. Differential Expression between DP and DN Monocytes in Late EVD, Related to Figure S6 Log fold-change, *p*-values, and *q*-values for differential expression tests comparing gene expression levels between DP and DN monocytes in late EVD. Also includes annotations of whether each gene is associated with macrophage differentiation, interferon response, or cell cycle.

Table S6. Association between Host Gene Expression and Viral Load for Infected Monocytes *In Vivo* and *Ex Vivo*, Related to Figure 7 Log fold-change, *p*-values, and *q*-values for differential expression tests of association between log10 viral load and gene expression for cells containing ≥ 1 viral read. Tab 1 is for *in vivo* (cells in DPI 5-8), and tab 2 is for *ex vivo* (cells treated with live virus and collected at 24 hours post inoculation). Tabs 3 and 4 show the gene set enrichment results for separate Fisher’s exact tests of genes that are significantly positively or negatively correlated with intracellular viral load (p < 0.05) including enrichment odds ratios (OR), p values, and q-values.

Data S1. Comparison of Unsupervised Clustering and Manual Gating of CyTOF Data, Related to Figure 2 CyTOF gating strategy used to define cell populations using canonical protein markers. We compared this approach against unsupervised clustering (Figure 2) and observed general agreement.
