# Supplementary material for: Single-Cell Profiling of Ebola Virus Disease In Vivo Reveals Viral and Host Dynamics
Source: Cell. 2020 Nov 25;183(5):1383–1401.e19. doi: 10.1016/j.cell.2020.10.002 (PMC7707107; doi:10.1016/j.cell.2020.10.002)
Supplement: Data S1. Comparison of Unsupervised Clustering and Manual Gating of CyTOF Data, Related to Figure 2 — CyTOF gating strategy used to define cell populations using canonical protein markers. We compared this approach against unsupervised clustering (Figure 2) and observed general agreement. [file mmc7.pdf]

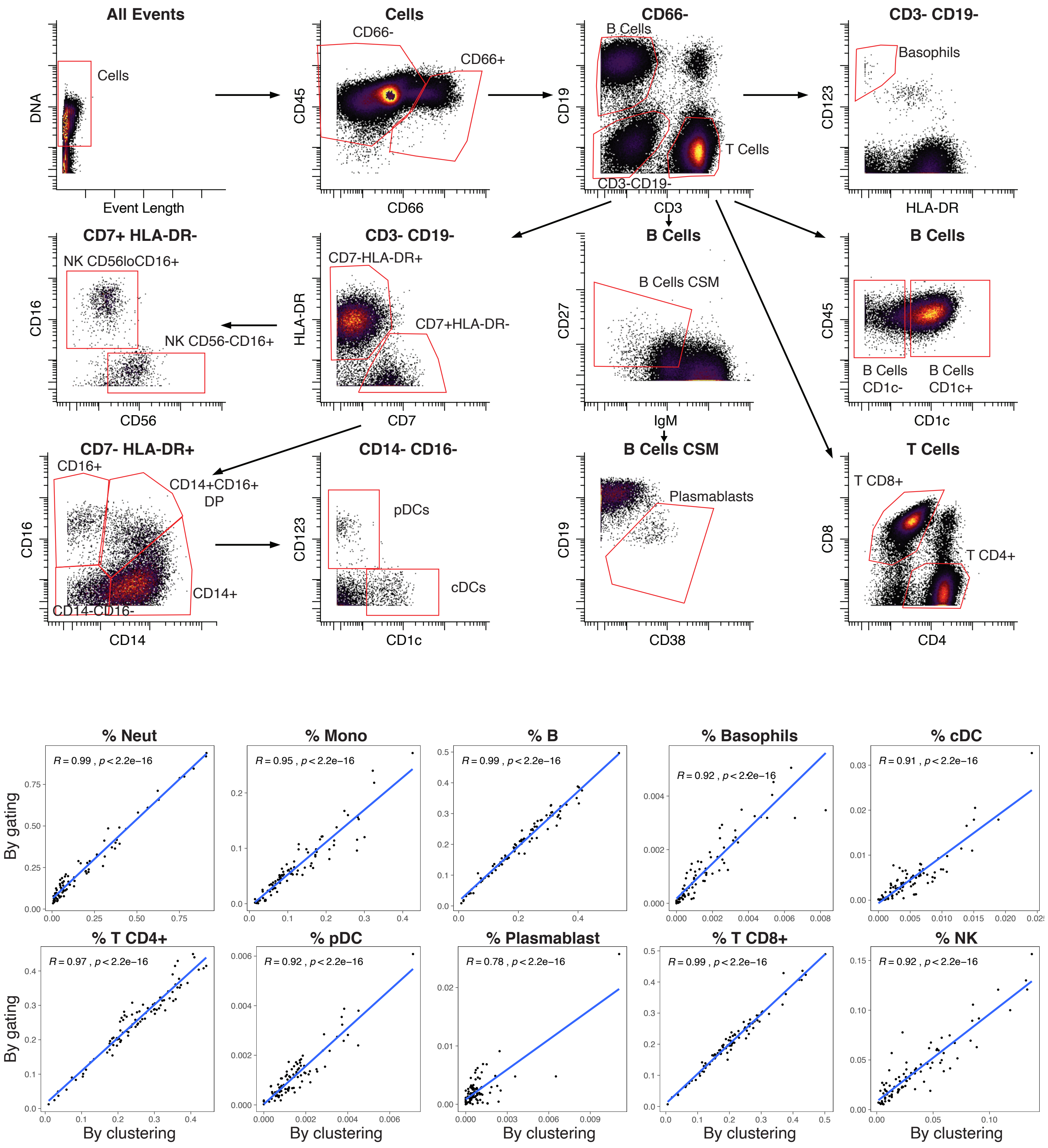

Manual gating agrees with unsupervised clustering of CyTOF data

Top: gating strategy used to define cell populations using canonical markers. Axes indicate CyTOF ArcSinh-scaled marker intensities.

Bottom: Pearson correlation between relative cell-type abundance determined by unsupervised clustering (x-axis) and by manual gating (y-axis). Each marker represents a single PBMC sample.

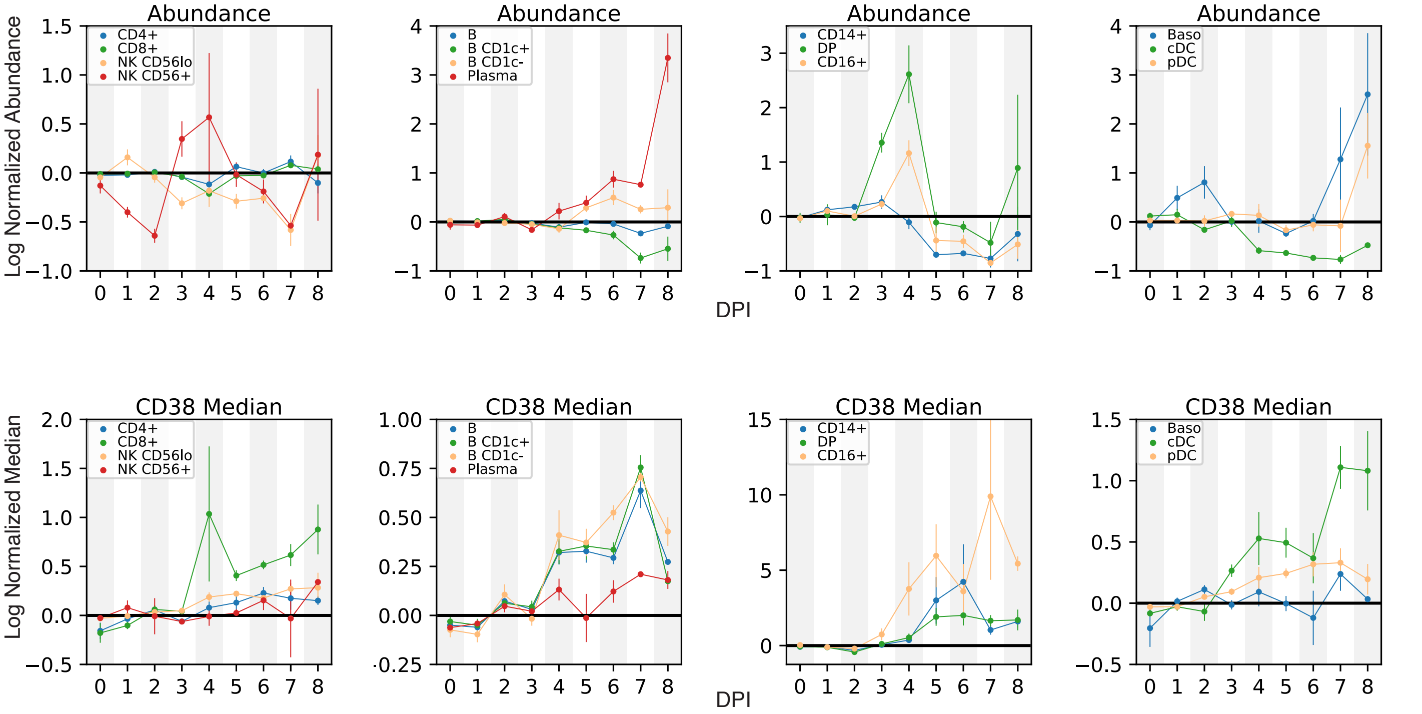

Manual gating and unsupervised clustering lead to similar cell-type abundances and activation

Top: Abundances of indicated manually gated populations, normalized for each animal to pre-challenge timepoints. x-axis shows day post infection (DPI), lines denote mean ± 1 standard error of the mean (SEM).

Bottom: Median CD38 expression on manually gated populations shown in (C), medians are normalized to each NHP's average baseline expression. x-axis shows DPI, lines denote mean ± 1 SEM.

Cocktail	Antigen	Clone	Vendor and Catalog	RRID	Label	Conc. (μg/mL)
Surface	CD38	AT-1	Abcam ab131420	AB_11158787	89	8.00
Surface	CD45	DO58-1283	BD 552566	AB_394433	115	5.37
Surface	CD233	BRIC 6	International Blood Group Reference Laboratory 9439		113	4.11
Surface	CD61	VI-PL2	Biolegend 336402	AB_1227584	140	5.37
Surface	CD66	YTH71.3	Thermo MA5-17003	AB_2538475	158	2.25
Surface	CD20	2H7	Biolegend 302302	AB_314250	173	5.37
Surface	IgM	G20-127	BD 555780	AB_396115	174	2.68
Surface	HLA-DR	Immu-357	Beckman Coulter		176	0.34
Surface	CD1c	AD5-8E7	Miltenyi		162	1.34
Surface	BDCA3	1A4	BD 559780	AB_397321	164	3.58
Surface	CD123	7G3	BD 554527	AB_395455	148	1.34
Surface	CCR7	150503	BD 561271	AB_10561679	FITC	8.94
Surface	CD3	SP34-2	BD 551916	AB_394293	157	2.68
Surface	CD4*	OKT4	Biolegend 317404	AB_571961	156	1.25
Surface	CD8	RPA-T8	Biolegend 301002	AB_314120	155	1.41
Surface	CD45RA	HI100	Biolegend 304102	AB_314406	166	2.68
Surface	CD11b	ICRF44	Biolegend 301312	AB_314164	153	6.71
Surface	CD11c	3.9	Biolegend 301602	AB_314172	143	1.34
Surface	CD14	M5E2	Biolegend 301810	AB_314192	151	10.73
Surface	CD16	3G8	Biolegend 302033	AB_2104002	159	2.68
Surface	CD33	AC104.3E3	Miltenyi		142	4.29
Surface	CD56	NCAM16.2	BD 559043	AB_397180	175	1.79
Surface	CD7	M-T701	BD 555359	AB_395762	141	5.37
Surface	CD161	HP-3G10	Biolegend 339902	AB_1501090	168	3.08
Surface	CD27	LT27	Biorad MCA755GA	AB_321554	209	3.00
Intracellular	STAT1 pY701	4a	BD 612232	AB_399555	147	3.83
Intracellular	STAT3 pY705	4/P-STAT3	BD 612357	AB_399646	139	5.82
Intracellular	STAT4 pY693	38/p-Stat4	BD 612738	AB_399957	170	5.66
Intracellular	STAT5 pY694	47/Stat5	BD 611965	AB_399386	149	5.37
Intracellular	STAT6 pY691	18/P-Stat6	BD 611567	AB_399013	165	5.38
Intracellular	Ki67	SolA15	Thermo 17-5698-82	AB_2688057	169	3.36
Intracellular	Erk1/2 pT202/Y204	D13.14.4E	CST 4370S	AB_2315112	152	16.44
Intracellular	MAPKAPK2 pT334	27B7	CST 3007S	AB_490936	144	2.29
Intracellular	CREB pS133	87G3	CST 9198S	AB_2561044	145	7.24
Intracellular	IkBa amino-terminal	L35A5	CST 4814S	AB_390781	163	3.79
Intracellular	TBK1/NAK pS172	D52C2	CST 5483S	AB_10693472	161	10.78
Intracellular	S6 pS235/236	2F9	CST 4858S		150	13.15
Intracellular	Zap70/Syk pY319/Y352	17A/P-ZAP70	BD 612575	AB_399864	160	2.14
Intracellular	4E-BP1 pT37/46	236B4	CST 2855S	AB_560835	172	3.13
Intracellular	PLCγ2 pY759	K86-689.37	BD		146	2.18
Intracellular	P38 pT180/Y182	36/p38	BD 612289	AB_399606	154	3.56
Intracellular	FITC (for CCR7)	FIT-22	Biolegend 408302	AB_528901	171	5.33
Intracellular	FoxP3	PCH101	Thermo 14-4776-82	AB_467554	167	10.55

Antibody panel for cell staining prior to CyTOF. Related to Key Resources Table.
See also (Bjornson-Hooper et al., 2019a, 2019b).
